# The Noonan Syndrome-linked *Raf1^L613V^* mutation drives increased glial number in the mouse cortex and enhanced learning

**DOI:** 10.1371/journal.pgen.1008108

**Published:** 2019-04-24

**Authors:** Michael C. Holter, Lauren. T. Hewitt, Stephanie V. Koebele, Jessica M. Judd, Lei Xing, Heather A. Bimonte-Nelson, Cheryl D. Conrad, Toshiyuki Araki, Benjamin G. Neel, William D. Snider, Jason M. Newbern

**Affiliations:** 1 School of Life Sciences, Arizona State University, Tempe, Arizona, United States of America; 2 Department of Psychology, Arizona State University, Tempe, Arizona, United States of America; 3 Arizona Alzheimer’s Consortium, Phoenix, Arizona, United States of America; 4 Neuroscience Center, The University of North Carolina School of Medicine, Chapel Hill, North Carolina, United States of America; 5 Laura and Isaac Perlmutter Cancer Center, New York University Langone Medical Center, New York, New York, United States of America; Stanford University School of Medicine, UNITED STATES

## Abstract

RASopathies are a family of related syndromes caused by mutations in regulators of the RAS/Extracellular Regulated Kinase 1/2 (ERK1/2) signaling cascade that often result in neurological deficits. RASopathy mutations in upstream regulatory components, such as *NF1*, *PTPN11/SHP2*, and *RAS* have been well-characterized, but mutation-specific differences in the pathogenesis of nervous system abnormalities remain poorly understood, especially those involving mutations downstream of *RAS*. Here, we assessed cellular and behavioral phenotypes in mice expressing a *Raf1*^*L613V*^ gain-of-function mutation associated with the RASopathy, Noonan Syndrome. We report that *Raf1*^*L613V/wt*^ mutants do not exhibit a significantly altered number of excitatory or inhibitory neurons in the cortex. However, we observed a significant increase in the number of specific glial subtypes in the forebrain. The density of GFAP^+^ astrocytes was significantly increased in the adult *Raf1*^*L613V/wt*^ cortex and hippocampus relative to controls. OLIG2^+^ oligodendrocyte progenitor cells were also increased in number in mutant cortices, but we detected no significant change in myelination. Behavioral analyses revealed no significant changes in voluntary locomotor activity, anxiety-like behavior, or sociability. Surprisingly, *Raf1*^*L613V/wt*^ mice performed better than controls in select aspects of the water radial-arm maze, Morris water maze, and cued fear conditioning tasks. Overall, these data show that increased astrocyte and oligodendrocyte progenitor cell (OPC) density in the cortex coincides with enhanced cognition in *Raf1*^*L613V/wt*^ mutants and further highlight the distinct effects of RASopathy mutations on nervous system development and function.

## Author summary

The RASopathies are a large and complex family of syndromes caused by mutations in the RAS/MAPK signaling cascade with no known cure. Individuals with these syndromes often present with heart defects, craniofacial differences, and neurological abnormalities, such as developmental delay, cognitive changes, epilepsy, and an increased risk of autism. However, there is wide variation in the extent of intellectual ability between individuals. It is currently unclear how different RASopathy mutations affect brain development. Here, we describe the cellular and behavioral consequences of a mutation in a gene called Raf1 that is associated with a common RASopathy, Noonan Syndrome. We find that mice harboring a mutation in Raf1 show moderate increases in the number of two subsets of glial cells, which is also observed in a number of other RASopathy brain samples. Surprisingly, we found that Raf1 mutant mice show improved performance in several learning and memory tasks. Our work highlights potential mutation-specific changes in RASopathy brain function and helps set the framework for future personalized therapeutic approaches.

## Introduction

The canonical RAS/RAF/MEK/ERK (aka ERK1/2 or MAPK3/MAPK1) intracellular signaling cascade is a crucial regulator of specific aspects of neural development and synaptic function [1,2,3,4,5,6,7,8]. Mutations that lead to altered ERK1/2 signaling give rise to a group of human developmental syndromes, commonly referred to as “RASopathies” [9]. Cardiac, craniofacial, and neurological abnormalities, such as developmental delay, hypotonia, intellectual/cognitive disability, and epilepsy, are often observed in individuals with RASopathies, in addition to an increased risk of autism [10,11,12,13]. Autism-like phenotypes and changes in ERK1/2 activity have also been detected in mouse models of Angelman (MIM: 105830), Rett (MIM: 312750), and Fragile X (MIM: 300624) syndromes [14,15,16,17,18]. The majority of RASopathy mutations lead to hyperactive signaling and are concentrated in classic components of Receptor Tyrosine Kinase (RTK)-linked intracellular signaling cascades. These include ‘upstream’ regulators of multiple cascades (*PTPN11* (MIM: 176876), *NF1* (MIM: 162200), *SHOC2* (MIM: 602775), *SOS1/2* (MIM: 182530, 601247), *SYNGAP1* (MIM: 603384), *SPRED1* (MIM: 609291), *SPRY1* (MIM: 602465), *K/N/HRAS* (MIM: 190070, 164790, 190020)), and relatively ‘downstream’ kinases in the core ERK1/2 pathway (*BRAF/RAF1* (MIM: 164757, 164760), *MEK1/2* (MIM: 176872, 601263), *ERK1/2* (MIM: 601795, 176948)) [11,19,20,21,22]. Individuals with Noonan Syndrome (MIM: 163950) comprise ~50% of all RASopathy cases and mutations have been observed in multiple genes, including *PTPN11/SHP2*, *SOS1*, *SHOC2*, *KRAS*, *NRAS*, *LZTR1* (MIM: 600574), *RAF1/CRAF*, and *MAP2K1/MEK1* [13,21,23]. While the genetic cause for most RASopathies is known, therapeutic options remain limited, due in part to an incomplete understanding of disease neuropathogenesis.

RASopathies are often associated with diminished intellectual functioning and neuropsychiatric impairment, but these phenotypes are highly variable [13,20,24,25,26]. Individuals with mutations in kinases downstream of RAS (e.g. *RAF*, *MEK*) generally exhibit more pronounced neurocognitive deficits in comparison to mutations in upstream regulators of RAS activity [20,26,27,28]. This is perhaps surprising since upstream mutations could lead to abnormalities in multiple parallel signaling cascades, including AKT/mTOR, RHO/ROCK, and PKA, in addition to ERK1/2 [29,30,31,32]. Pharmacological modulation of ROCK, NOTCH, PKC, or cAMP/PKA signaling appear to mitigate select cellular defects in animal models, indicating ERK1/2-independent contributions to RASopathy pathogenesis [29,30,33,34,35]. Nonetheless, animal model studies have demonstrated that pharmacological inhibitors of hyperactive ERK1/2 signaling reverse many RASopathy-linked phenotypes [1,5,36,37,38]. It is unclear whether ERK/MAPK inhibitors should be utilized in response to mutations that are linked to variable neurocognitive outcomes. For example, *RAF1*^*L613V*^ individuals mostly present with intellectual impairment [39,40,41], but *RAF1*^*L613V*^ individuals with normal IQ [42] and even increased IQ [43] have also been observed. Further study of mutated components downstream of RAS (i.e., RAF or MEK) might assist in defining the specific contributions of altered ERK1/2 signaling to the cellular and behavioral defects in RASopathies.

Studies of the developmental effects of ERK1/2 and RASopathy mutations in model organisms have identified alterations in embryonic stages of neurogenesis and gliogenesis [6,44,45,46,47,48,49]. Abnormalities in cortical neuron morphology and synaptic plasticity during postnatal periods have also been implicated in learning deficits [1,4,5,8,30,46,48,50,51,52];. ERK1/2 activation in neurons promotes specific forms of synaptic plasticity and learning [52]. When expressed selectively in mature glutamatergic neurons, the Costello Syndrome-associated *Hras*^*G12V+/-*^ mutation enhanced phosphorylated-ERK1/2 (p-ERK1/2) levels, increased LTP, and resulted in improved performance in spatial learning and contextual fear conditioning [4]. However, germline *Hras*^*G12V*^ mice do not exhibit differences in LTP and display diminished spatial learning capabilities [53]. These findings hint at distinct contributions of neuronal vs non-neuronal changes to plasticity and learning in the RASopathies that likely vary at different stages of development.

Glial cells are critical for the proper formation, maturation, and plasticity of neural circuits [54]. ERK1/2 signaling is an important mediator of gliogenesis, glial proliferation, and function [6,44,55,56,57,58,59,60]. For example, a hallmark feature of post-mortem Neurofibromatosis Type 1 (NF1) (MIM: 162200) patient forebrains and mouse models of NF1 is an increased number of GFAP^+^ reactive astrocytes [60,61,62,63]. Additionally, diffusion tensor imaging (DTI) analyses have identified white matter differences in individuals with NF1 and the autism-linked 16p11.2 microduplication (MIM: 614671), which includes ERK1 [64,65]. Collectively, these data speak to an important, yet poorly understood role for astrocyte and oligodendrocyte dysfunction in RASopathy neuropathogenesis.

Here we studied the establishment of neuronal and glial number and behavior in mice heterozygous for a *Raf1*^*L613V*^ variant linked to Noonan Syndrome in humans [38,39,40]. *Raf1*^*L613V/wt*^ heterozygous mice exhibit embryonic lethality on a pure C57Bl/6J background but survive on a mixed C57Bl/6J x 129S1 background with notable deficits in cardiac and craniofacial development [38,66]. Even though Noonan Syndrome is associated with a range of neurological abnormalities, nervous system development has not been evaluated in *Raf1*^*L613V/wt*^ mouse mutants. Here, we report increases in GFAP^+^-astrocyte and OLIG2^+^-oligodendrocyte-progenitor cell (OPC) density in the mature forebrains of *Raf1*^*L613V/wt*^ mice without a significant difference in cortical neuron density. Remarkably, *Raf1*^*L613V/wt*^ mutant mice exhibit moderate enhancements in spatial reference memory, spatial working memory, and fear learning tasks. Taken together, our data show that the Noonan Syndrome-linked *Raf1*^*L613V/wt*^ mutation increases the number of two glial subtypes and enhances distinct aspects of cognition.

## Materials and methods

### Ethics statement

All experimental designs using mice were reviewed and approved by the Institutional Animal Care and Usage Committees at the University of North Carolina Chapel Hill and Arizona State University (Protocol#17-1521R). All mice examined in this study were euthanized via CO2 inhalation or fully anesthetized with avertin prior to transcardial perfusion as described in the AVMA Guidelines on Euthanasia.

### Mice and genotyping

The generation of mice harboring the *Raf1*^*L613V*^ knock-in mutation has been previously described [38]. Due to embryonic lethality when backcrossed onto the C57Bl/6J genetic background [38], these experiments utilized mice maintained on a C57Bl/6J x 129S1 mixed background (JAX Stock # 101043). Mice were housed under standard laboratory conditions with *ad libitum* access to food and water on a 12-hour light/dark cycle in vivaria at UNC and ASU. For PCR genotyping, we utilized a single primer set to amplify a 560bp fragment of the *Raf1*^*L613V*^ allele and a 500bp wild-type allele from genomic DNA samples (Forward (5’-3’): AGTCAGCCTAGAGGCCACTGTTA, Reverse (5’-3’): CTCCAATTTTCACCGTGAGGC).

### Tissue preparation and immunohistochemistry

Mice were fully anesthetized and transcardially perfused with cold 4% PFA in 1X PBS. Brains were then dissected, post-fixed overnight, mounted in a 3% agarose block, and sectioned on a EMS 7000smz-2 vibrating microtome. For most experiments, brains from a littermate control/mutant pair were sectioned, immunolabeled, and imaged in parallel. Tissue sections were collected in PBS, permeabilized in 0.2% Triton X-100 in PBS, and incubated in a blocking solution consisting of 5% Normal Donkey Serum and 0.2% Triton X-100 in PBS. Primary antibodies were then diluted in blocking solution and incubated with tissue sections at 4C for 24–48 hours while rocking gently. The primary antibodies used were: rabbit anti-p-ERK (1:1000, Cell Signaling 4370), mouse anti-RBFOX3/NeuN (1:1000, Millipore MAB377), goat anti-Parvalbumin (1:1000, Swant PVG-214), rabbit anti-GFAP (1:1000, Abcam ab7260), rabbit anti-ACSBG1 (1:500, Abcam ab65154), mouse anti-S100β (1:1000, Sigma SAB1402349), rabbit anti-IBA1 (1:1000, Wako 019–19741), rabbit anti-OLIG2 (1:1000, Millipore AB9610), rat anti-MBP (1:1000, Abcam ab7349), rabbit anti-PDGFRα (1:1000, Santa Cruz sc-338), rabbit anti-NG2 (1:500, Millipore AB5320), mouse anti-CC1 (1:500, Calbiochem), rabbit anti-Arc (1:1000, Synaptic Systems 156 003), rabbit anti-TNFα (1:2000, Bio-Rad), and biotinylated WFA (20μg/mL, Vector). Tissue sections were then washed in 0.2% Triton in PBS and incubated in fluorescently-conjugated secondary antibodies including donkey anti-rabbit, donkey anti-rat, donkey anti-mouse, and donkey anti-goat IgGs conjugated to Alexa Fluor 488, 555, 568, or 647 dyes (Invitrogen). For WFA labeling, a streptavidin-conjugated 488 secondary antibody was used. Brain slices were then slide mounted, coverslipped in Fluoromount (EMS #17984), and stored at 4C prior to imaging.

### Electron microscopy

P60 mice were anesthetized, transcardially perfused with 2% paraformaldehyde, 2% glutaraldehyde in 0.1M PB and post-fixed overnight. A 3mm x 1mm x 1mm block was sub-dissected from the genu of the corpus callosum for myelination assessment. Tissue blocks were then washed 3x with PB before a 2 hr secondary fixation with 1% osmium tetroxide in 0.1M PB. Tissue blocks were washed 3x with deionized water and stored at 4C overnight. The next day, the tissue blocks were returned to room temperature and dehydrated in a series of three acetone washes, increasing in 20% increments per wash. The tissue blocks were then infiltrated with Spurr’s epoxy resin three times at increasing increments of 25% pure resin, and sectioned in a Leica Ultracut-R microtome at a section thickness of 70nm, stained with 2% uranyl acetate in 50% ethanol for six minutes, and then moved to Sato’s lead citrate for four minutes. Genu sections were visualized in a Philips CM12 TEM at 80kV, and images were captured with a Gatan model 791 slow-scan CCD camera in the Biological Electron Microscopy Facility at Arizona State University.

### Image analysis and quantification

For immunofluorescent analyses, images were collected on a Leica SP5 or Zeiss LSM800 confocal microscope from at least three different tissue sections per mouse and at least three mice per group. Confocal optical sections for quantification are typically collected from a z-axis region between 5–15 μm from the surface of the tissue section. Following optimization of image brightness and contrast, regions of interest (ROI) were outlined in images of anatomically matched sections using standard neuroanatomical boundaries. The number of labeled cells was determined by a blinded observer in at least three individual ROIs per mouse spanning all layers of a cortical or hippocampal column contained within a specified sub-region. The number of cells was divided by the area of the ROI and averaged across all ROIs from an individual brain to estimate the density of labeled cells in a single mouse brain. To calculate relative density, the average density was normalized to the age-matched littermate control processed in parallel. Results were analyzed for statistical significance using the Students t-test.

For myelination analysis, electron micrographs within a cross-section of the genu of the corpus callosum were assessed for axon area, g-ratio, and the proportion of myelinated to unmyelinated axons. Axon g-ratios were calculated as the cross-sectional diameter of the axon excluding the myelin sheath, divided by the total diameter of the axonal fiber including the myelin sheath. The numbers of myelinated and unmyelinated axons were counted within a given image, and the proportion of myelinated to unmyelinated axons was calculated for mutants and controls. Results are reported as the average ± SEM and compared using the Student’s t-test.

### Western blotting

Cortices were dissected and lysed in RIPA buffer (0.05M Tris-HCl, pH 7.4, 0.5M NaCl, 0.25% deoxycholic acid, 1% NP-40, and 1mM EDTA, Millipore), supplemented with 0.1% SDS, protease inhibitor cocktail (Sigma) and phosphatase inhibitor cocktails II and III (Sigma). Lysates were cleared by centrifugation, and protein concentration was determined. Equal amounts of protein were denatured in reducing sample buffer, separated by SDS-PAGE gels, and transferred to PVDF membranes (Bio-Rad). Blots were blocked with 5% BSA in TBS containing 0.5% Tween 20 (TBS-T) for 1 h at room temperature, then incubated overnight at 4°C with primary antibodies. The primary antibodies used were: rabbit anti-p-ERK1/2 (Thr202/Tyr204) (Cell Signaling Technology, Inc), rabbit anti-ERK1/2 (CST), rabbit anti-MEK1/2 (Ser217/221) (CST), rabbit MEK1/2 (CST), rabbit anti-DUSP6 (Abcam ab76310), rabbit anti-SPRY2 (Abcam ab85670), and anti-GAPDH (Cell Signaling Technology, Inc.). After washing with TBS-T, membranes were incubated with HRP-conjugated secondary antibodies in 5% milk in TBS-T for 2 h at room temperature. Blots were washed with TBS-T, and detection was performed with SuperSignal West Pico chemiluminescent substrate (Thermo Scientific).

### Behavior

All behavior experiments were performed at ASU with mice kept on a standard light cycle in a room dedicated to behavioral assessment. The experimenter was blinded to the mouse genotype during animal testing and data analysis. No statistically significant difference was observed between male and female mice; therefore, results were pooled for presentation.

### Open field, elevated plus, and social approach assay

The open field, elevated plus, and social approach tests were performed on 23 control (6 male, 17 female) and 31 mutant (16 male, 15 female) mice with at least three days between different behavioral assays. The open field was used to test voluntary locomotive and anxiety-like behaviors. The apparatus consisted of a 40x40cm arena enclosed by 30cm high opaque walls. A single 60W bulb was positioned to brightly illuminate the center of the chamber with dim lighting near the walls. Mice were placed into the apparatus and video recorded for a total of 10 minutes. Video data were analyzed for distance traveled and time spent in the center quadrant.

The elevated plus maze was constructed from black Plexiglas, elevated 81cm off the ground, and oriented in a plus formation with two 12x55cm open arms and two 12x55cm closed arms extending from an open 12x12cm center square. Closed arm walls were 40cm high extending from the base of the arm at the center square. The apparatus was lit with a 60W bulb with light concentrated on the center square. At the beginning of each trial, mice were placed in the center square, facing the south open arm, and recorded while freely exploring for 5 minutes.

The social approach apparatus contained three 20x30x30cm chambers (total dimensions 60x30x30cm) connected by open doorways. Prior to experimental social trials, mice were habituated to the apparatus and allowed to freely explore all three chambers for 5 minutes. At the end of the 5 minutes, mice were removed and placed in their home cage. A sex- and age-matched stimulus mouse was then placed into a small empty cage in chamber 1 of the apparatus. The experimental mouse was reintroduced to the center chamber (chamber 2) of the apparatus and recorded while freely exploring for 10 minutes. The time spent in the chamber with the stimulus mouse (chamber 1) or the empty chamber (chamber 3) was then measured.

Video recordings of the open field, elevated plus, and social approach tests were collected and quantified in ImageJ using publicly available plugins [67]. followed by statistical analysis using the Students t-test.

### Water radial-arm maze

The water radial-arm maze (WRAM) was used to evaluate spatial working and reference memory [68,69]. The maze consisted of an eight-arm apparatus (each arm 38.1 × 12.7cm) filled with opaque, room temperature water. Water temperature was consistently between 18–20°C for testing. Extra maze spatial cues were present to aid mice in spatial navigation. In the win-shift version of WRAM, mice (n = 19 control, 15 mutant, all female) were required to find hidden platforms (10 cm diameter) submerged at the end of four out of the eight arms. Platform location patterns were assigned to each subject and stayed constant for a particular mouse across all testing days, but varied among subjects. Mice received four trials per day for 18 days.

Trials began with the subject being released from the start arm and given 2 min to locate a platform. Arm entries were manually recorded when the mouse’s body crossed the threshold of the mouth of the arm. Once a platform was found, the animal remained on it for 15 seconds and was returned to its heated testing cage for a 30s inter-trial interval (ITI). During the ITI, the just-found platform was removed from the maze, and the water was cleaned to remove any debris and obscure olfactory cues. The mouse was then placed back into the start arm and given another 2 min trial to locate a platform. Because a platform is removed from the maze for the remainder of the day after discovery, mice must maintain several items of information in order to effectively solve the task, thus increasingly taxing the working memory system as trials progress within a day. The number of arm entries into non-platformed arms is quantified as errors as the dependent measure of spatial memory in the task [68]. Errors are further divided into particular error types. Working Memory Correct (WMC) errors are defined as all entries into arms that previously contained a platform within a day. Reference Memory (REF) errors are first entries into arms that never contained a platform, and Working Memory Incorrect (WMI) errors are all subsequent entries into arms that never contained a platform within a day. WMC, REF, and WMI errors are summed for a total error score. All errors were quantified using orthogonal measures of working and reference memory, as previously reported [68,69]. Data were analyzed using Statview statistical software with a repeated measures ANOVA followed by Fisher’s LSD *post-hoc* test, when indicated.

### Morris water maze

Five days after the last day of WRAM testing, spatial reference memory was evaluated in the same cohort of mice using the Morris water maze (MM) (n = 19 control, 15 mutant, all female). The apparatus was a round tub (188cm diameter) filled with opaque, room temperature water (18–20°C), and contained a submerged platform (10cm diameter) in the northeast quadrant. The platform location remained fixed across all days and trials, with spatial cues available to aid the animals in spatial navigation, testing spatial reference memory (Morris et al., 1982). Mice received four trials per day for five days. At the beginning of each trial, mice were placed into the tub from one of four starting points (north, south, east or west). The order of the drop-off location varied semi-randomly across days, but was the same within a day for all subjects. MM performance was recorded using a video camera and tracking system (Ethovision; Noldus Instruments; Wageningen, The Netherlands). Mice had 60s to locate the platform, where they remained for 15s before being placed back into a heated cage for an ITI of 5–8min. A probe trial was given on the fifth day of testing, during which the platform was removed and mice were given 60s to swim freely in the maze. Following the first probe trial day, a reversal task was carried out for two consecutive days. Specifically, the platform location was moved from the northeast quadrant to the opposite, southwest quadrant, where it remained across all reversal task trials. Mice then received four trials per day for two days. A second probe trial followed the last baseline trial on day two of the reversal task. For acute behavioral stimulation of Arc expression, a separate subset of mice (n = 4 mutants, 4 controls) were placed in the Morris maze for 5-trials with 15 minutes between trials in one day and sacrificed 60 minutes after the end of the 5^th^ trial for immunohistochemical analyses. Data were analyzed using Statview statistical software with a repeated measures ANOVA followed by Fisher’s LSD *post-hoc* test, when indicated.

### Visible platform task

After completion of cognitive behavioral testing on the WRAM and MM, mice were evaluated using the visible platform control task to assess locomotor and visual performance. The apparatus was a rectangular tub (100 × 60cm) filled with clear room temperature water (18–20°C). A black platform (10cm wide) was positioned 4cm above the surface of the water. A ring of opaque curtains surrounded the tub, blocking all obvious spatial cues. Animals received three trials in one day. The drop off location remained the same across trials; however, the platform location varied semi-randomly across three locations. Each mouse had 90s to locate the platform, where it remained for 15s before being placed back into a heated cage for an ITI of 5–8min.

### Fear conditioning

Control (n = 11, 3 male, 8 female) and *Raf1*^*L613V/wt*^ (n = 9, 2 male, 7 female) adult mice were placed in test cages (12"Wx10"Dx12"H: Coulbourn Instruments, E10-18TC) housed within a sound-attenuating cabinet (Coulbourn, E10-23, white, 31.5” W x 21” D x 20” H) with an attached video camera that recorded behavior for offline scoring of all fear conditioning procedures. Seventy-five (75) dB tones of 30 second duration were produced by a frequency generator (Coulbourn, E12-01 or H12-07) and delivered through a speaker (Coulbourn, H12-01R) on the side panel. Video recordings were analyzed for the number of seconds mice spent freezing, a species-typical fear response that is defined by the absence of all movement except those associated with respiration, during the 30 sec prior to, and during, tone presentation. An animal shock generator (Coulbourn, H13-15) produced an electrical current (0.25mA, 1 sec) evenly delivered to metal bars in the cage flooring (Coulbourn, E10-11R/M-TC-SF). All stimuli were controlled using Graphic State software (v 3.0) installed on a computer connected to a stimuli output controller system (Coulbourn, H02-08).

Training and testing were performed in two distinct contexts (A and B) with varying rooms and investigator appearance. Context A had silver, metal walls on the sides, clear Plexiglas front and back with yellow paper located outside, metal bar shock floor, white drop pan, and cleaned with grapefruit scented cleaner (Method, Target Inc.). For testing in context B, the chambers had walls covered with vertical black and white striped inserts, a cross-hatched non-shock metal grid floor, black drop pans, and cleaned with 70% isopropyl alcohol. Mice were acclimated to the testing room for 20 min and to each of the two contexts for 10 min on the three days prior to training. In the training session for Context A, mice were presented with three trials of an auditory tone that co-terminated with presentation of a foot shock. Twenty-four (24) and 48 hours later, mice were tested for memory by undergoing three tone-only trials in context B at each time point. Mice then underwent an extinction paradigm where tones were repeatedly presented (maximum of 16) until total freezing was less than 10 seconds during tone presentation. One week later, spontaneous recovery was assessed by presenting them with three presentations of the tone to determine whether freezing to tone was due to associative or non-associative processes. After this testing was complete, we performed a shock intensity test, where shocks were gradually increased from a minimum of 0.08mA until the mouse elicited a clear startle response. Statistical analysis was conducted using Students t-test or repeated measures ANOVA, followed by *post-hoc* tests in SPSS.

## Results

### Cortical neuron number is normal in Raf1^L613V/wt^ mutant mice

**Fig 1:**
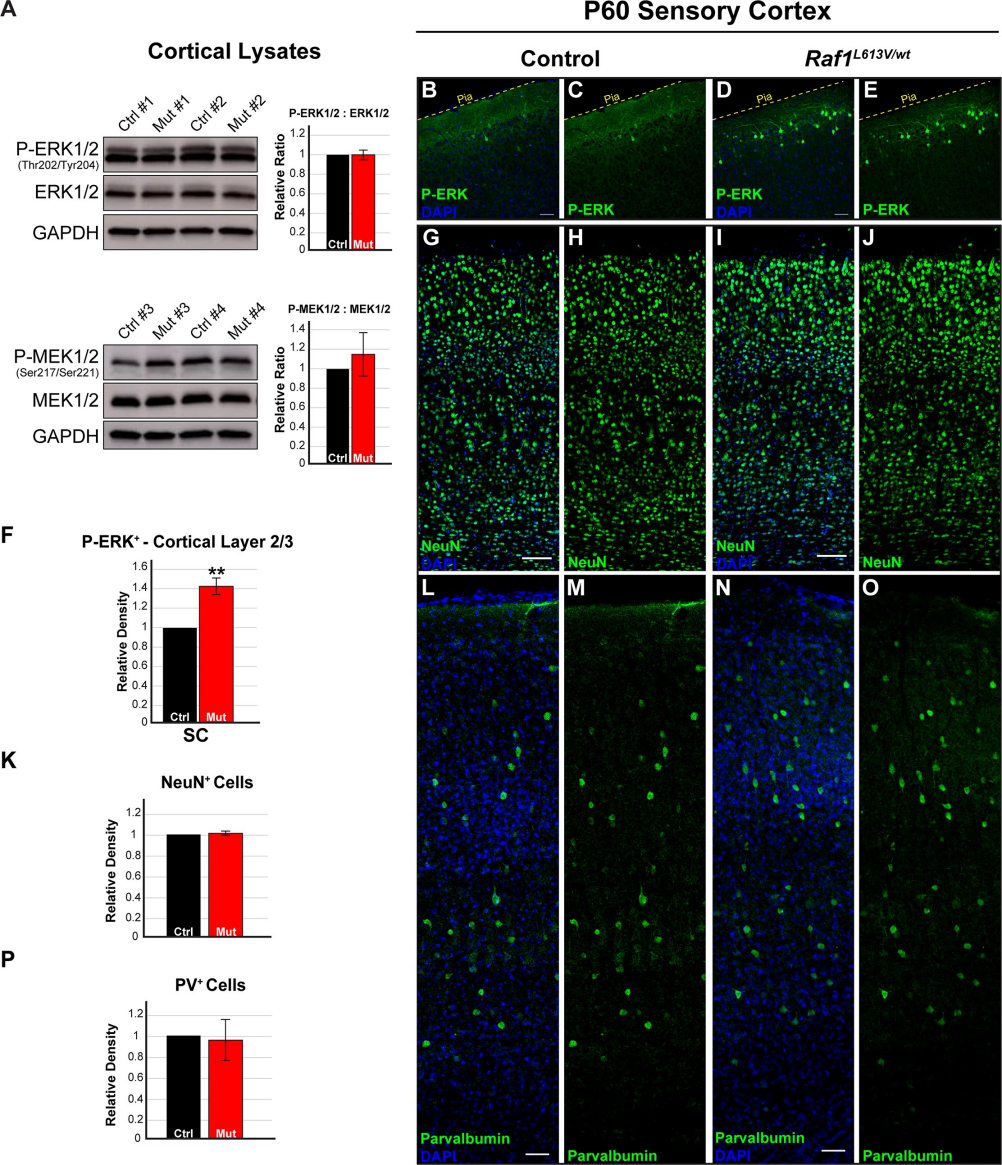
*Raf1*^*L613V/wt*^ mutants do not exhibit significant alterations in cortical neuron density. **A**: Western blots of P21 whole cortical lysates from *Raf1*^*L613V/wt*^ cortices exhibit no relative change in the total levels of MEK1/2 or ERK1/2 or the phosphorylation of MEK1/2 or ERK1/2, as measured by the ratio of phosphorylated to total protein (mean ± SEM, n = 5). **B-F:** Representative confocal images of p-ERK1/2 immunolabeling in adult (P60) sensory cortex reveals an increased density of p-ERK1/2-expressing pyramidal cells in mutant layer 2 (D-E), compared with littermate controls (B-C); quantification in F (mean ± SEM, n = 3, p < 0.01). **G-K:** Representative immunolabeled sections of sensory cortex showing NEUN-labeled neurons and DAPI-labeled nuclei. *Raf1*^*L613V/wt*^ (I-J) cortical NEUN^+^ density does not significantly differ from controls (G-H), as quantified in K (mean ± SEM, n = 3). **L-P:** Quantification of Parvalbumin-labeled cortical GABAergic neurons in control (L-M) and mutant (N-O) cortices reveals no significant alterations in cell density (P) (mean ± SEM, n = 3). All scale bars = 50μm.

We generated *Raf1*^*L613V/wt*^ heterozygous mice on mixed C57Bl/6J x 129S1 background. Past work has shown that embryonic and cardiac fibroblasts from *Raf1*^*L613V/wt*^ mice do not exhibit a difference in basal ERK1/2 activity but show a significant stimulus-dependent increase in levels of p-ERK1/2 following treatment with different RTK-linked trophic cues, including EGF, FGF2, PDGF, and IGF-1 [38,66]. Western blot assessment of whole cortical lysates revealed no relative difference in basal p-MEK, MEK, p-ERK1/2 or total ERK1/2 levels between *Raf1*^*L613V/wt*^ and control adult mice (Fig 1A, n = 5). Moreover, we detected no change in the negative feedback regulators SPRY2 and DUSP6 in cortical lysates from *Raf1*^*L613V/wt*^ and control mice (S1A Fig; n = 5). *Raf1*^*L613V/wt*^ and control mice also showed comparable patterns of hippocampal p-ERK1/2 labeling (S1B and S1C Fig). A subpopulation of layer 2 cortical excitatory neurons receives high levels of glutamatergic input, a known activator of ERK1/2 signaling, and expresses increased levels of FOS, an immediate early gene product regulated, in part, by ERK1/2 activity [52,70]. In agreement with previous work, we noted many p-ERK1/2-labeled neurons in layer 2/3 of sensory cortex in adult control mice [71,72,73]. However, mutant mice exhibited a significantly larger number of p-ERK1/2 labeled neurons in layer 2, but not deeper layers, of the sensory cortex compared to age-matched controls (Fig 1B–1E, F; n = 4, p < 0.01). These data are consistent with the anatomically restricted, local increases in p-ERK1/2 observed in *HRas*^*G12V*^ mice [53], and suggest that the *Raf1*^*L613V*^ mutation drives an increase in stimulus-dependent ERK1/2 signaling in a subset of endogenously active cortical neurons.

ERK1/2 signaling in radial glia regulates the trajectory of cortical neurogenesis and cortical excitatory neuron number [6,48,50]. We assessed the number of cells in *Raf1*^*L613V/wt*^ and control cortices expressing a well-established neuronal marker, RBFOX3/NeuN, which is highly expressed in nearly all excitatory neurons in the cortex (Fig 1G–1J). The relative density of RBFOX3/NeuN^+^ neurons in the adult sensory cortex was unchanged, suggesting no significant alterations to neurogenesis (Fig 1K; n = 3). GABAergic neurons comprise ~20% of the total neuron population in the cortex and express relatively low levels of RBFOX3/NeuN. We immunolabeled for parvalbumin (PV) and somatostatin (SST) to assess the density of the two largest cortical GABAergic neuron subpopulations. PV-expressing GABAergic neurons were distributed normally in cortical layers with no significant alterations in density (Fig 1L–1O, P; n = 3). Taken together, our data indicate that the *Raf1*^*L613V/wt*^ mutation does not alter the distribution and number of mature excitatory and inhibitory neurons in the adult cortex.

### Increased density of GFAP^+^ astrocytes in Raf1^L613V/wt^ forebrains

**Fig 2:**
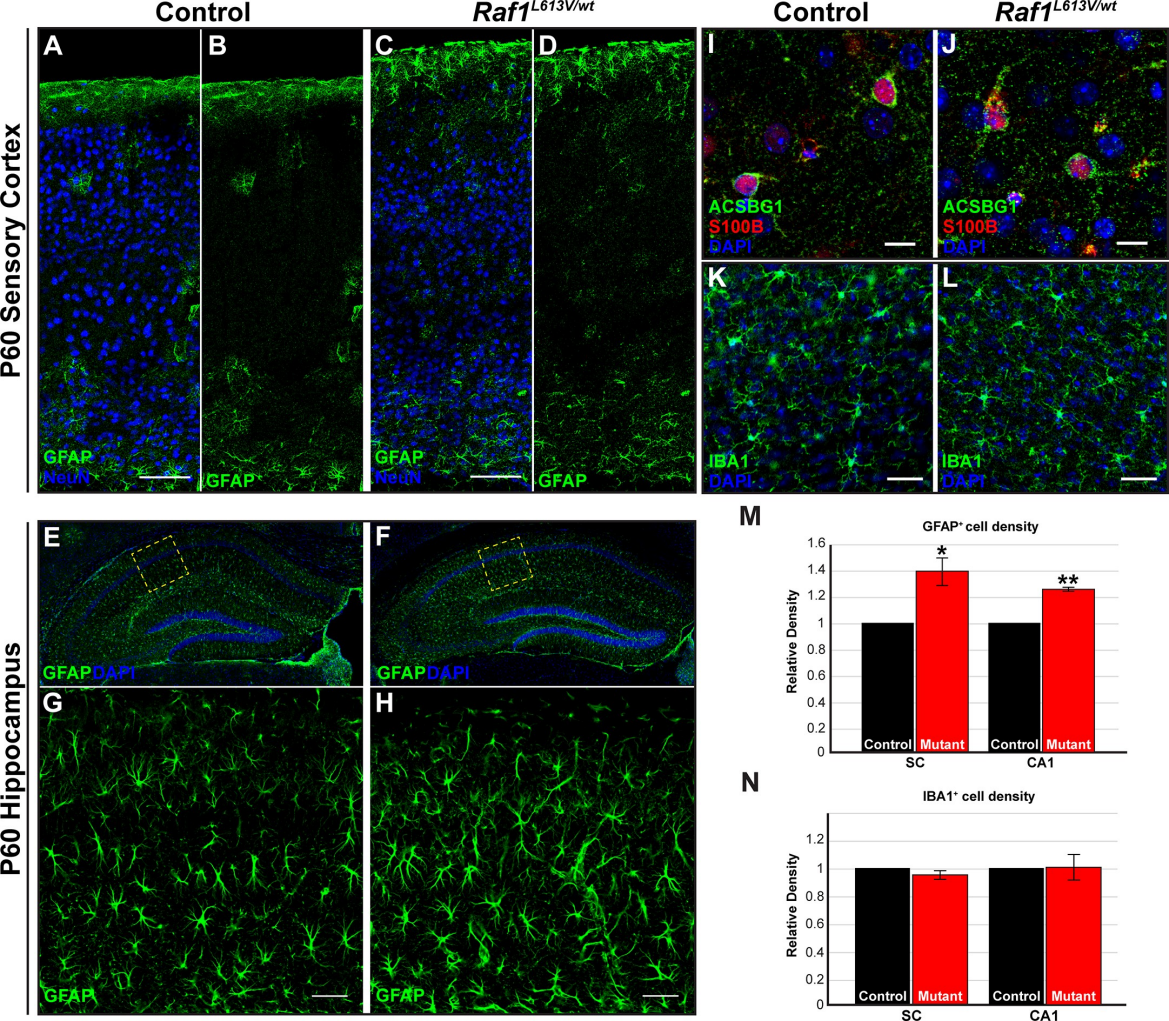
Increased density of GFAP^+^ astrocytes, but not IBA1^+^ microglia, in *Raf1*^*L613V/wt*^ cortex and hippocampus. **A-D:** Representative confocal images of GFAP-immunolabeled control (A-B) or mutant (C-D) sensory cortices show a significantly increased density of GFAP^+^ astrocytes across cortical layers (M) of mutant mice (mean ± SEM, n = 3, *p < 0.05) (scale bar = 100μm). **E-F:** Coronal cross-sections of control (E, inset in G) and *Raf1*^*L613V/wt*^ (F, inset in H) hippocampus following immunostaining for GFAP. Mutant hippocampal CA1 sub-regions demonstrate a significantly increased relative density of GFAP^+^ astrocytes, relative to littermate controls (M) (mean ± SEM, n = 3, *p < 0.01) (scale bar = 50μm). **I-J:** Triple-labeled representative images of control (I) and mutant (J) cortex following staining for the grey matter astrocyte marker ACSBG1, pan-glial marker S100β, and DAPI (n = 3). **K-L, N:** No significant change in the density of microglia in the cortex is observed in mutant mice (L) relative to controls (K); quantification in N (mean ± SEM, n = 3) (scale bar = 25um).

Altered glial number and function are observed in response to RASopathy-linked *Nf1*, *Ptpn11/Shp2*, and *Ras* mutations [6,57,59,60,61,62]. To determine if *Raf1*^*L613V/wt*^ mutant mice display alterations in glial development, we immunolabeled for glial fibrillary acidic protein (GFAP) and Acyl-CoA synthetase bubblegum family member 1 (ACSBG1), markers of fibrous and protoplasmic astrocytes, respectively, and the glial marker S100β [74]. Astrocytes expressing these canonical markers were clearly labeled in adult forebrains from control and mutant mice (Fig 2A–2J). In control cortices, GFAP^+^ astrocytes were enriched in the white matter, upper layer 1–2, and layer 6, though labeled profiles were occasionally detectable in the remaining cortical layers. We found a significantly increased density of GFAP^+^ astrocytes in *Raf1*^*L613V/wt*^ sensory cortices across all layers, relative to littermate controls (Fig 2A–2D, M; n = 3, p < 0.05). In comparison to the cortex, GFAP^+^ astrocytes are present at a relatively higher abundance throughout the wild-type hippocampus. Assessment of the GFAP^+^ astrocyte population in the CA1 region of the mutant hippocampus also revealed an increase in density (Fig 2E–2H, M; n = 3, p < 0.01) in mutant mice. These data demonstrate that GFAP^+^ astrocyte number is increased in *Raf1*^*L613V/wt*^ mutants across multiple brain regions.

In states of injury, gliosis is often characterized by an increased density of GFAP^+^ astrocytes, which usually coincides with microglial activation and proliferation [54]. We therefore labeled for the activated microglia marker, IBA1, to assess the effects of *Raf1*^*L613V/wt*^ in the cortex and hippocampus. We found that the density and distribution of IBA1^+^ activated microglia were evenly distributed across the cortical grey matter and did not significantly differ in density in mutants relative to littermate controls (Fig 2K, 2L and 2N; n = 3). Additionally, no change in the relative density of activated microglia was observed in the mutant hippocampus (Fig 2N; n = 3). In summary, the *Raf1*^*L613V/wt*^ mutation is sufficient to induce an increased density of GFAP^+^ astrocytes, but not neurons or microglia.

### Raf1^L613V^ drives enhanced oligodendrocyte progenitor cell density in the cortical grey and white matter but does not alter myelination

**Fig 3:**
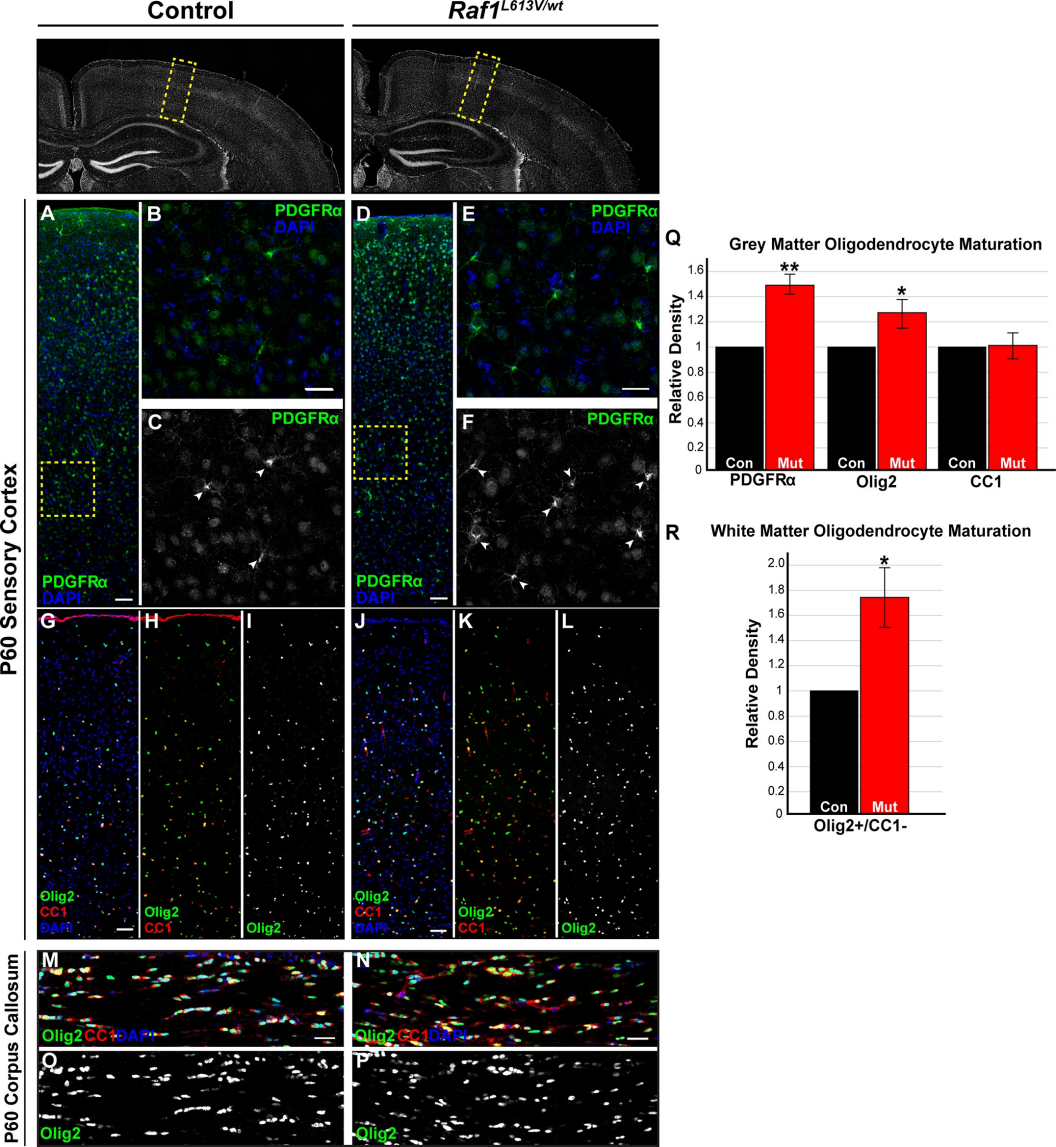
*Raf1*^*L613V/wt*^ mutants show increased numbers of OPCs but not myelinating oligodendrocytes. **A-F**: Representative immunolabeled sections of control (A, inset in B-C) and mutant (D, inset in E-F) sensory cortices reveal a significant relative increase in the number of PDGFRα^+^ cells in mutant mice (white arrow heads, mean ± SEM, n = 3, *p < 0.01). **G-L:** Triple-labeled representative images of staining for the pan-oligodendrocyte marker, Olig2, mature myelinating marker, CC1, and DAPI. Note the increased density of Olig2^+^ cells in the *Raf1*^*L613V/wt*^ sensory cortex (J-L), relative to controls (G-I), as quantified in M (mean ± SEM, n = 7, *p < 0.01). By contrast, analysis of CC1^+^ cells reveals no changes in the number of presumably mature, myelinating oligodendrocytes (Q) (mean ± SEM, n = 4). **M-P:** Assessment of Olig2^+^/CC1^-^ oligodendrocyte lineage cells revealed a significant increase in the density in the mutant (N, P) medial corpus callosum relative to age-matched controls (M, O) (R; n = 5, p < 0.05). All scale bars = 50μm.

Past work has linked hyperactive ERK1/2 signaling to alterations in oligodendrocyte development, including proliferation, differentiation, and myelination [3,75,76]. We first assessed the density of OPCs expressing PDGFRα in the adult *Raf1*^*L613V/wt*^ cortex. Immunolabeling for PDGFRα revealed an increased density of OPCs in the adult sensory cortex (Fig 3A–3F and 3M; n = 3, p < 0.01). PDGFRα is downregulated by mature oligodendrocytes; thus, we also examined the relative number of cells expressing Olig2, an independent marker of the oligodendrocyte lineage. We found increased numbers of Olig2^+^ cells in the cortical grey matter of adult *Raf1*^*L613V/wt*^ mutant mice (Fig 3I, 3L and 3M; n = 7, p < 0.01), but not the hippocampus (control relative density 1; mutant relative density 1.06; p = 0.66; n = 3). Olig2 is expressed by oligodendrocyte progenitor cells (OPCs) and mature oligodendrocytes. We distinguished between myelinating and immature oligodendrocyte lineage cells by co-labeling with the mature myelinating oligodendrocyte marker, CC1 [77]. As expected, all CC1^+^ cells present in the cortical grey matter co-expressed Olig2, but a subpopulation of Olig2^+^ cells did not express detectable levels of CC1, consistent with immature OPCs. *Raf1*^*L613V/wt*^ brains also had significantly increased densities of Olig2^+^/CC1^-^ cells in the corpus callosum as compared to littermate controls (Fig 3M–3P and 3R; n = 5, p < 0.05). Even though there was an increase in oligodendrocyte progenitors, we detected no significant difference in the density of CC1-labeled mature myelinating oligodendrocytes in *Raf1*^*L613V/wt*^ mutant cortices (Fig 3H, 3K and 3Q; n = 3).

**Fig 4:**
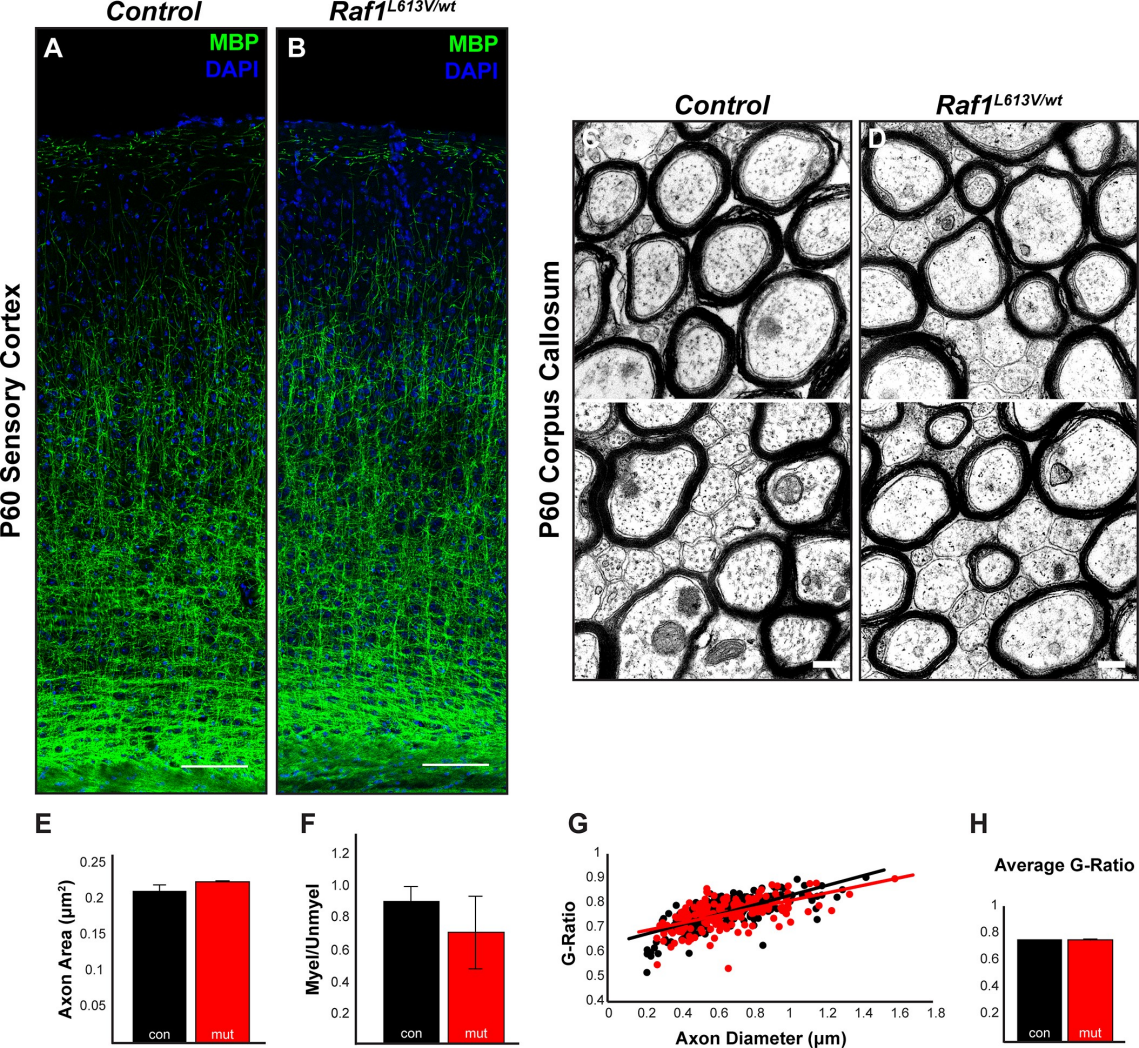
*Raf1*^*L613V/wt*^ cortices display normal myelination patterns. **A-B:** MBP-labeled regions of adult sensory cortices reveal no qualitative differences in the extent or pattern of myelination (n = 3) (scale bar = 50μm). **C-D:** Representative electron micrographs of the genu of the corpus callosum in control (C) and mutant (D) mice reveal no difference in axon area (E) or the proportion of myelinated to unmyelinated axons (F) (mean ± SEM, n = 3) (scale bar = 200nm). **G-H:** Myelination, as quantified by g-ratio, was not significantly different in mutant mice relative to controls (mean ± SEM, n = 3).

Strong gain-of-function ERK1/2 signaling drives increased myelination in adult animals [3]. However, the pattern of myelin basic protein (MBP) immunolabeling in P60 *Raf1*^*L613V/wt*^ cortices was indistinguishable from that of controls (Fig 4A and 4B). We also observed no difference in cortical MBP immunolabeling between mutants and controls at P14, an early stage of cortical myelination (S2A–S2D Fig). To quantify myelin thickness, we examined myelinated axons in the genu of the corpus callosum by electron microscopy (Fig 4C and 4D). We did not detect a significant change in average axon area (Fig 4E; n = 3) or the proportion of myelinated to unmyelinated axons (Fig 4F; n = 3) in adult *Raf1*^*L613V/wt*^ mutants. Moreover, we did not observe a significant difference in g-ratio, a normalized measure of myelin thickness that takes into account differences in axon diameter (Fig 4G and 4H; n = 3). Collectively, these data indicate an increased number of OPCs in the mutant cortex without alterations in mature oligodendrocyte number or myelination.

**Fig 5:**
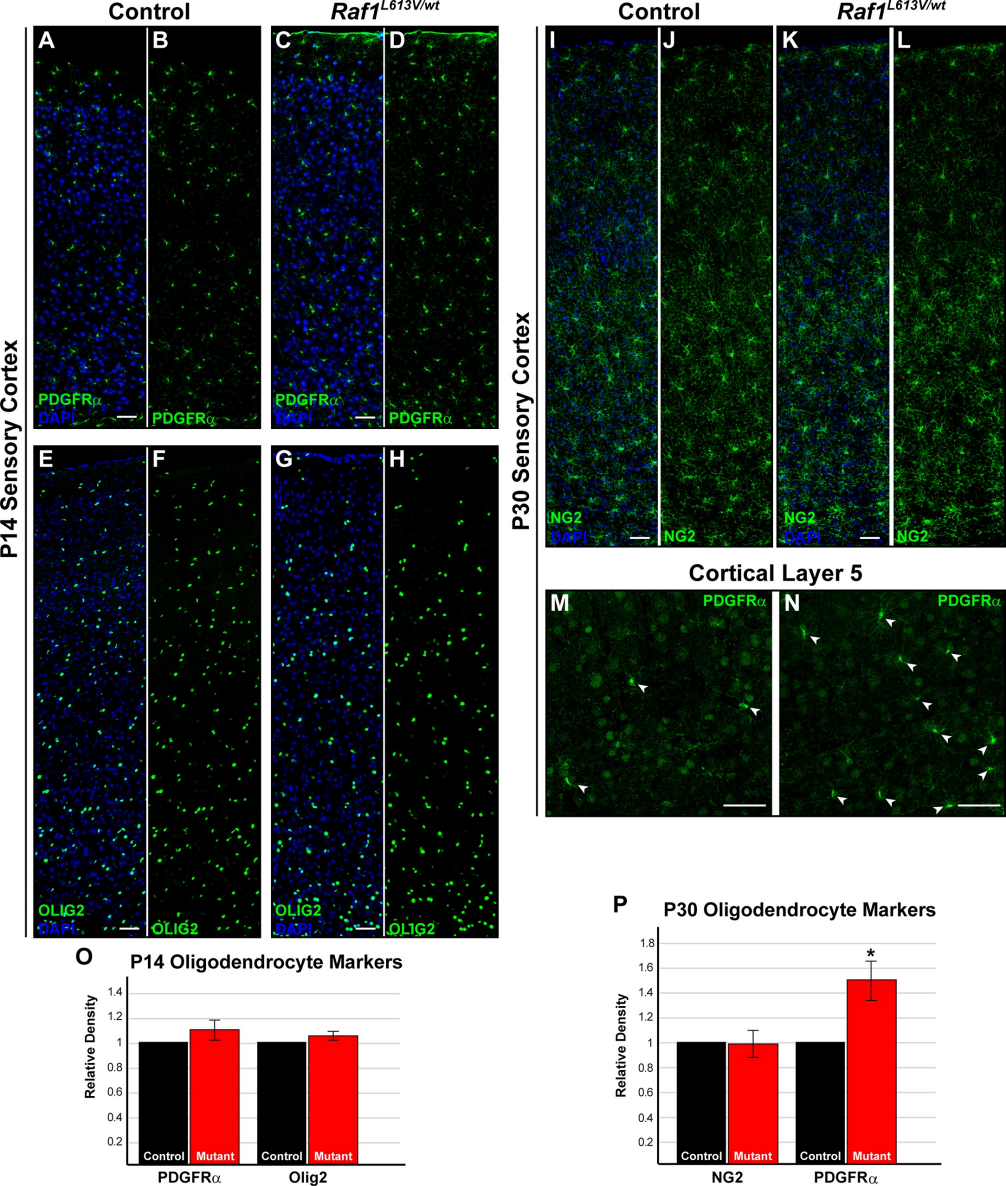
P14 mutant cortices do not display increased densities of PDGFRα^+^ or OLIG2^+^ cells. **A-H:** Sensory cortices from P14 control (A-B, E-F) and *Raf1*^*L613V/wt*^ (C-D, G-H) mice, immunolabeled for PDGFRα (A-D) or OLIG2 (E-H), show no relative difference in OPC cell density (O) (mean ± SEM, n = 3). **I-L:** Immunolabeling for an alternate marker of OPCs, NG2, in P30 control (I-J) and mutant (K-L) brains also results in no changes in relative density of labeled cells (P) (mean ± SEM, n = 3). **M-N:** PDGFRα immunolabeling (arrows) revealed a significant increase in the number of OPCs in mutant cortices in comparison to controls (P) (mean ± SEM, n = 3). All scale bars = 50μm.

To better understand the timing of increased OPC density in the adult cortex, we examined the cortical OPC pool in juvenile, P14 *Raf1*^*L613V/wt*^ mice. Immunolabeling for PDGFRα (Fig 5A–5D; n = 3) and Olig2 (Fig 5E–5H; n = 3) revealed no significant differences in OPC number in the cortex between P14 mutant mice and littermate controls (Fig 5O). Analysis of NG2-labeled, presumptive oligodendrocyte progenitors at P30 similarly yielded no significant difference in density (Fig 5I–5L, P; n = 3). However, we detected a significant increase in the density of PDGFRα-expressing cells at P30, indicating that the OPC pool expansion occurs post-adolescence (Fig 5M, 5N and 5P; n = 3; p < 0.05).

### Raf1^L613V/wt^ animals exhibit enhanced learning and memory

Individuals affected by the *Raf1*^*L613V*^ mutation present with variable levels of intellectual function [39,40,41,42,43]. The neurobehavioral characteristics of *Raf1*^*L613V/wt*^ mutant mice are unknown. RASopathies are often associated with behavioral phenotypes seen in autism, such as increased anxiety, decreased sociability, and locomotor impairments. We therefore exposed *Raf1*^*L613V/wt*^ mutant mice to a behavioral battery to assess these behaviors, as well as learning and memory performance.

**Fig 6:**
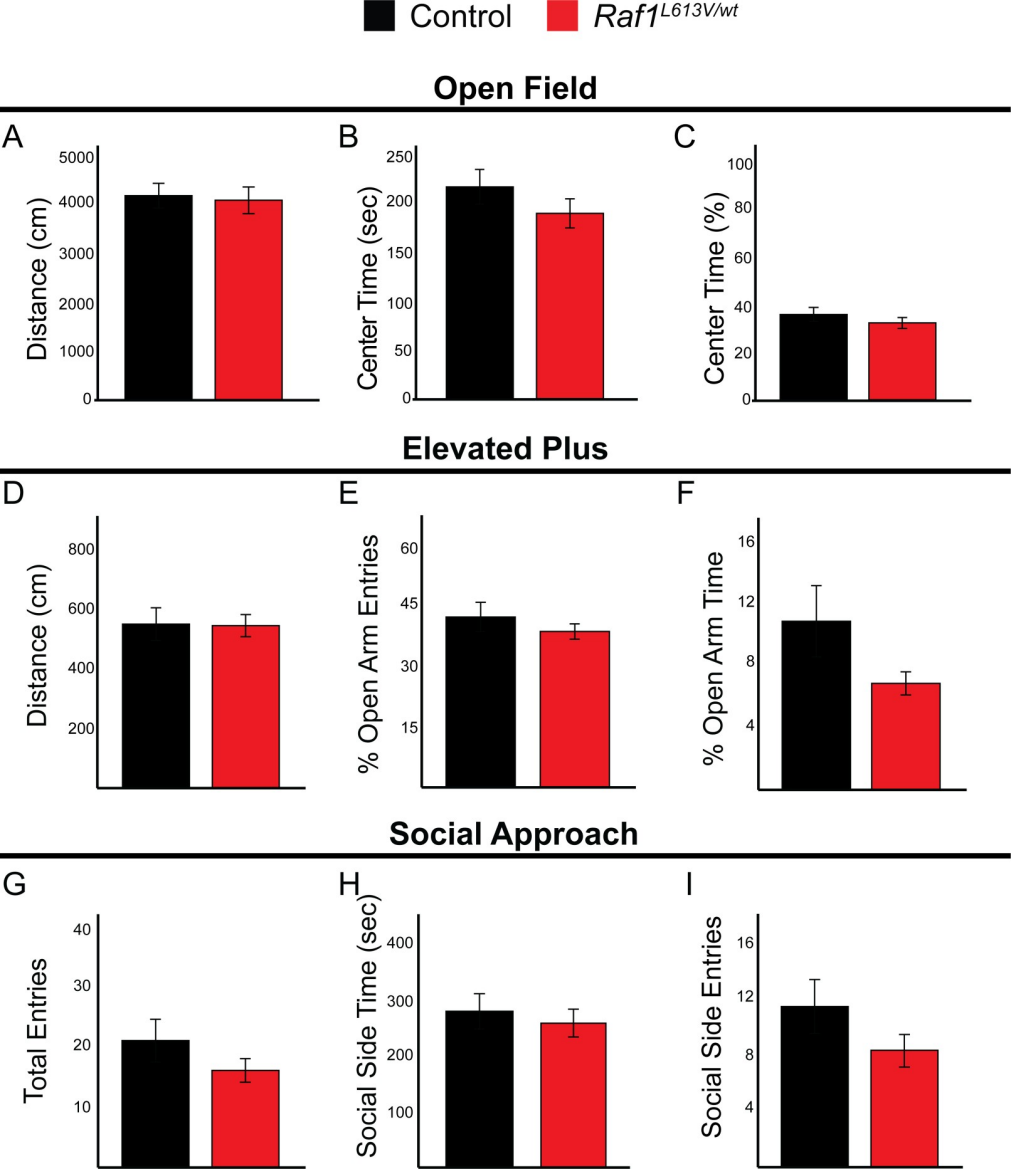
*Raf1*^*L613V/wt*^ mice have normal locomotor capabilities, anxiety-like behaviors, and sociability. Control (n = 23) and *Raf1*^*L613V/wt*^ (n = 31) mice were tested on a battery of behavioral tasks including the open field, elevated plus, and social approach assays. **A-C:** In the open field test, controls and *Raf1*^*L613V/wt*^ mice do not exhibit a difference in total distance traveled (**A**), center time (**B**) and percent center time (**C**) (mean ± SEM). **D-F:** Elevated plus maze testing of controls and *Raf1*^*L613V/wt*^ mice reveals that total distance (**D**), percent open arm entries (**E**), and open arm stay time (**F**) are unaltered in mutant mice (mean ± SEM). **G-I:** In the social approach task, controls and *Raf1*^*L613V/wt*^ mutants display similar performance, as measured by total entries (**G**), time in social side (**H**), and entries on social side (**I**) (mean ± SEM).

Adult *Raf1*^*L613V/wt*^ mutant displayed no abnormalities in three distinct tasks meant to assess anxiety-like behaviors, sociability, and locomotion compared to wild-type mice (Fig 6) [78,79]. In the open field assay, mutant mice did not display a significant difference in total distance traveled (Fig 6A) or time spent in the center quadrant, demonstrating no deficit in voluntary locomotor ability or anxiety-like behavior (Fig 6B and 6C; n = 23 controls, 31 mutants). These data were consistent with a separate test of locomotor activity and anxiety, the elevated plus maze, where no significant change in locomotor activity (Fig 6D) or time spent in the open arm was detected (Fig 6E and 6F; n = 23 controls, 31 mutants). Finally, the social approach assay did not reveal a significant difference between mutant and control mice in total entries (Fig 6G) or the time spent exploring the chamber with a novel mouse, indicating no deficit in sociability (Fig 6H and 6I; n = 21 controls, 31 mutants).

**Fig 7:**
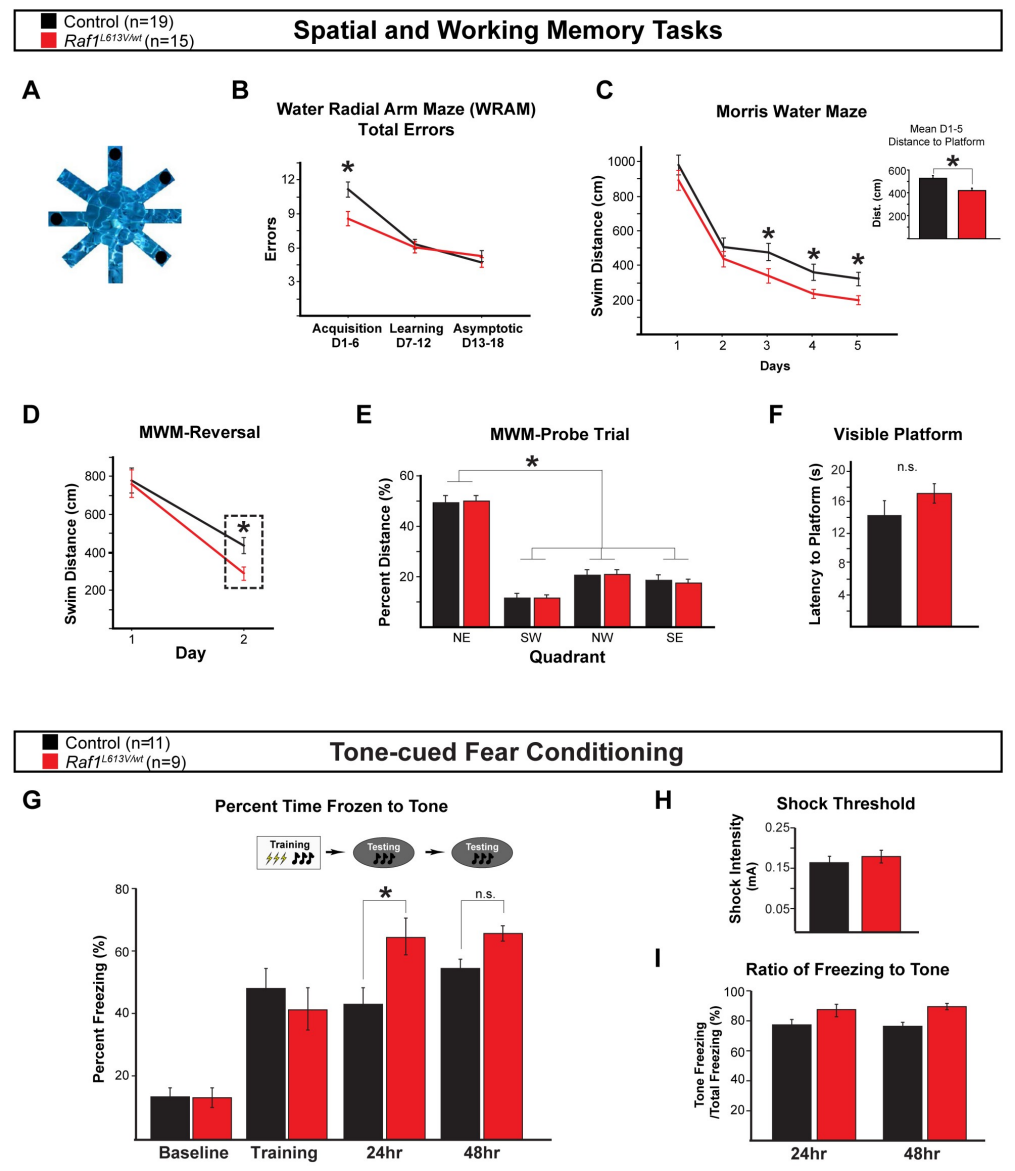
*Raf1*^*L613V/wt*^ animals exhibit enhanced spatial and working memory and fear learning. **A:** Schematic of an 8-armed water radial-arm maze (WRAM) used to examine adult control (n = 19) and *Raf1*^*L613V/wt*^ (n = 15) mice. **B:** During the acquisition phase of WRAM testing between days 1–6, mutants execute significantly fewer total errors than controls (mean ± SEM, main effect of genotype [F_(1, 32)_ = 4.87, p < 0.05] * = Fisher’s PLSD p < 0.05). **C:** In the Morris water maze (MWM), *Raf1*^*L613V/wt*^ mice show a reduction in swim distance to platform on days 3, 4, and 5 relative to controls (mean ± SEM, * = one-tailed t-test, p < 0.05). The inset graph shows the average across all 5 days of testing (mean ± SEM, [F_(1, 32)_ = 4.59, p < 0.05] * = Fisher’s PLSD p < 0.05). **D:** In the Morris maze reversal task, *Raf1*^*L613V/wt*^ mice demonstrate reduced swim distance to platform on day 2 (mean ± SEM, * = one-tailed t-test, p < 0.05). **E:** Probe trial analysis demonstrated significant preference for the NE target quadrant (mean ± SEM, [F_(3, 96)_ = 89.90, p < 0.0001] * = Fisher’s PLSD p < 0.0001). **F:** In the visible platform task, we did not detect a significant effect of genotype (mean ± SEM, [F_(1, 32)_ = 1.31, p = 0.26]). **G:** Naïve control (n = 11) and mutant (n = 9) animals were tested in tone-cued fear conditioning. Mutant animals do not differ significantly from controls in freezing behavior during the baseline or training phase; however, *Raf1*^*L613V/wt*^ mice spent a significantly greater amount of time frozen to tone 24-hours post training (mean ± SEM, [F_(3,54)_ = 3.429, p < 0.05] * = Fisher’s LSD post-hoc p < 0.05). No effect was detected 48-hours post training (mean ± SEM, Fisher’s LSD post-hoc p = 0.109). **H:** No significant differences in shock threshold were detected (mean ± SEM). **I:** Ratio of freezing to tone was unchanged at 24- and 48-hours post-training (mean ± SEM).

We next asked if *Raf1*^*L613V/wt*^ mutant mice displayed altered performance in an eight-armed water radial-arm maze (WRAM) swim task (Fig 7A). Control (n = 19) and mutant (n = 15) mice both showed a statistically significant decrease in the number of total errors over 18 days of testing (main effect of day [F_(17, 544)_ = 9.87, p < 0.0001]). Surprisingly, however, *Raf1*^*L613V/wt*^ mutant mice committed significantly fewer total errors in the acquisition phase (days 1–6) in comparison to controls (Fig 7B, main effect of genotype [F_(1, 32)_ = 4.87, p < 0.05] * = Fisher’s PLSD p < 0.05). When errors were assessed by type, mutant mice made fewer working memory correct (WMC) errors, related to reentering an arm that previously had a platform within a day (S3A Fig) (main effect of genotype [F_(1, 32)_ = 8.94, p < 0.01] * = Fisher’s PLSD p < 0.01). Moreover, we detected a marginal trial by treatment interaction for WMC errors during the acquisition phase, indicating that as working memory load increases, mutants tended to make fewer WMC errors than controls (S3B Fig) (marginal interaction of trial by genotype [F_(2, 64)_ = 2.83, p = 0.07], Trial 3 Only: [F_(1, 32)_ = 6.83, p < 0.05], Trial 4 Only: [F_(1, 32)_ = 5.82, p < 0.05]). Neither working-memory incorrect (WMI) nor reference memory (REF) errors significantly differed between genotypes during the acquisition phase (S3C Fig; WMI [F_(1, 32)_ = 2.05, p = 0.16], REF [F_(1, 32)_ = 0.41, p = 0.53]). We did not observe significant differences between genotypes during the learning and asymptotic phases, once mice have successfully acquired the task and solve the maze at peak performance (i.e. asymptotic phase) (Fig 7B; learning: [F_(1, 32)_ = 0.05, p = 0.82]; asymptotic: [F_(1, 32)_ = 2.26, p = 0.14]).

Five days following the completion of WRAM testing, the same cohort of mice was tested in the Morris water maze. Control and *Raf1*^*L613V/wt*^ mutant mice exhibited improved performance over time (Fig 7C, main effect of day [F_(4, 128)_ = 71.58, p < 0.0001]). However, mutant mice displayed enhanced performance relative to controls, as measured by swim distance to platform across all five days of testing (Fig 7C [F_(1, 32)_ = 4.59, p < 0.05] inset * = Fisher’s PLSD post-hoc p < 0.05). Analysis of individual days revealed that mutant mice performed better than controls on days 3, 4, and 5, as predicted by the enhanced performance during WRAM acquisition (Fig 7C, * = one-tailed t-test, p < 0.05). Mutant mice also demonstrated reduced swim distance to the platform on day 2 of reversal learning (Fig 7D; * = one-tailed t-test, p < 0.05). Controls and mutants both displayed the expected target quadrant preference, as indicated by a significantly higher percent of total swim distance in the previously platformed quadrant during the probe trial for baseline and reversal task testing (Fig 7E, S3D and S3E Fig). Additionally, mutants and controls did not exhibit significant differences in the visible platform test, indicating intact visual and motoric capacity (Fig 7F).

We employed a tone-cued fear conditioning paradigm to examine amygdala-dependent learning and memory. Control (n = 11) and mutant (n = 9) mice were placed in a testing cage fitted with a shock floor, and given a 30-second auditory tone, immediately followed by a foot-shock, for a total of three tone-foot shock pairings. Control and mutant mice displayed similar shock thresholds (Fig 7H) and baseline time freezing during the 30-second tone immediately prior to the first foot shock pairing (Fig 7G). Control and mutant mice both exhibited an increase in average time freezing during the subsequent two tone presentations of the training phase (Fig 7G; main effect of trial [F_(3, 54)_ = 36.47, p < 0.001] LSD post-hoc p < 0.001). No difference in freezing between genotypes was detected during training (Fig 7G; [F_(1, 18)_ = 0.59, p = 0.45]). Twenty-four (24) and 48-hrs after acquiring the tone-foot shock association, mice were placed in a different context and tested for freezing behavior in response to the auditory tone without the paired foot shock. *Raf1*^*L613V/wt*^ mice displayed significantly increased time freezing to tone in comparison to controls 24-hrs post-training (Fig 7G; interaction between genotype and trial ([F_(3,54)_ = 3.43, p < 0.05] * = LSD post-hoc p < 0.05), but not at 48-hrs (Fisher’s LSD post-hoc p = 0.11). A significant difference in time spent freezing to tone was not observed during extinction learning or in spontaneous recovery seven days post-extinction (Fig 7I, S3F Fig). Taken together, *Raf1*^*L613V/wt*^ mice demonstrate moderately enhanced performance in hippocampal-dependent spatial working and reference memory tasks and amygdala-dependent fear memory.

### Alterations in regulators of synaptic plasticity are not detectable in Raf1^L613V/wt^ mutant mice

**Fig 8:**
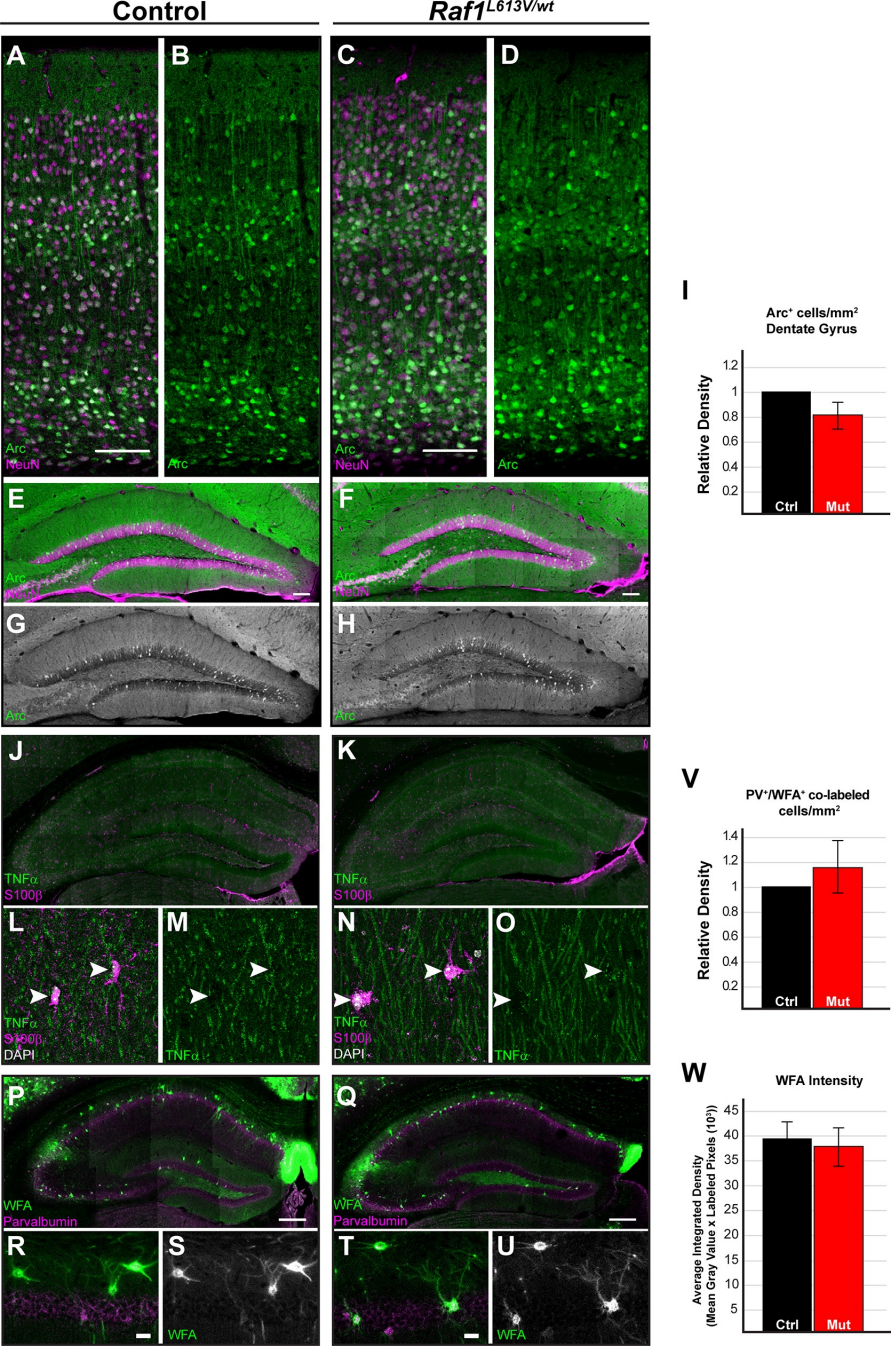
Normal expression of markers of synaptic plasticity in *Raf1*^*L613V/wt*^ neurons and astrocytes. **A-H:** Control and mutant mice were subjected to a single 5-trial day of Morris Water Maze testing and collected 1-hour after completion of the 5^th^ trial (n = 4). **A-D:** Double immunolabeled sections of sensory cortex for the activity-dependent gene ARC and mature neuronal marker NEUN. We did not detect any differences in the pattern of Arc labeling in the cortex. (scale bar = 100 μm) **E-I:** Counts of labeled cells in the granular layers of the dentate gyrus reveal no change in the density of ARC^+^ cells (mean ± SEM, n = 4). **J-W:** Assessment of markers of astrocyte function in behaviorally naïve mice. **J, K:** Representative coronal sections of hippocampus immunolabeled for TNFα and S100β. **L-O:** Confocal micrographs (63x) of TNFα- and S100β-labeled astrocytes indicates no qualitative change in the levels of TNFα in mutant hippocampi (n = 3). **P, Q:** Double immunolabeled coronal sections of hippocampus, stained with PV and the perineuronal net marker WFA (scale bar = 250μm). **R-U:** Representative images of CA1 hippocampal WFA-labeled neurons show no significant differences in the extent of perineuronal net formation (scale bar = 25μm). **V:** No significant differences in the number of WFA-labeled PV cells are seen (mean ± SEM, n = 3). **W:** Mutant (n = 35) and control (n = 37) PV neurons have a similar extent of surrounding WFA-labeled pixels (mean ± SEM).

To identify the mechanisms that drive enhanced behavioral performance in learning and memory assays in *Raf1*^*L613V/wt*^ mice, we assessed the expression of ARC, TNFα, and perineuronal net components, known regulators of synaptic plasticity that have also been linked to altered ERK1/2 signaling [52,80]. We subjected behaviorally naïve adult mice to five trials in the Morris water maze to activate the hippocampal circuit. Brains were collected 1 hour after the final trial and the number of ARC^+^ cells were assessed in the cortex and hippocampus. *Raf1*^*L613V/wt*^ mice did not exhibit significant differences in ARC expression in comparison to controls in the cortex (Fig 8A–8D, n = 4), or in the granule cell layer of the dentate gyrus (Fig 8E–8I, n = 4).

We also examined the expression of putative astrocyte-derived modulators of synaptic plasticity. The astrocyte-secreted molecule, TNFα, is a major contributor to learning and memory [80,81,82], and astrocyte-mediated perineuronal net formation around PV^+^ GABAergic neurons is thought to contribute to RASopathy phenotypes[59]. We therefore assessed behaviorally-naïve *Raf1*^*L613V/wt*^ mice for the expression of TNFα and a perineuronal net marker, *wisteria floribunda* agglutinin (WFA), in the hippocampus. Qualitative analysis of hippocampal TNFα revealed no apparent changes to the pattern of expression in the hippocampal region globally or in mutant S100βw^+^ glia (Fig 8J–8O). We next examined the extent of WFA-labeling surrounding PV^+^ GABAergic neurons within the CA1 region of the hippocampus (Fig 8P–8U). We found that *Raf1*^*L613V/wt*^ PV^+^ GABAergic neurons within the hippocampus displayed a similar extent and amount of WFA-labeling in comparison to controls (Fig 8P–8U, W; n = 35 mutant and 37 control neurons from 3 independent pairs). Therefore, *Raf1*^*L613V/wt*^ mutant mice do not exhibit significant alterations in the expression of these three mediators of synaptic plasticity.

## Discussion

Our study provides insight into the cellular and behavioral processes affected by a Noonan Syndrome-linked *Raf1*^*L613V*^ mutation. In contrast to RASopathy-linked *PTPN11* or *RAS* mutants, we found little evidence that *Raf1*^*L613V*^ expression significantly alters global cortical excitatory or PV-expressing inhibitory neuron number [44,46,83]. However, we detected an increased density of GFAP^+^ astrocytes in the mutant cortex and hippocampus [6,44,45,46,47,48,49,84]. OPC number was also increased in mutant cortices, though no significant change was observed in mature oligodendrocyte density or myelination. The increase in select glial subtypes did not correlate with a significant difference in locomotor ability, anxiety, or sociability. In contrast to many NF1 and Noonan Syndrome mouse models, *Raf1*^*L613V/wt*^ mice displayed moderately enhanced performance in three different learning and memory tasks. Overall, these data show that the ‘downstream’ RASopathy-linked *Raf1*^*L613V*^ mutation increases the number of GFAP^+^ astrocytes and OPCs and improves aspects of learning and memory without significant alterations in basal behavioral measures of anxiety or sociability.

Pharmacological inhibitors of RAS and MEK1/2 reverse several aspects of nervous system dysfunction in many RASopathy rodent models [5,38,51,85]. However, clinical trials with pathway inhibitors, primarily statins in NF1 patients, have had limited success, possibly due to ERK1/2-independent pathways modified in response to mutations at the level of or upstream of RAS [29,30,32,58,86,87,88,89,90]. Defining the effects of mutations at multiple levels of the cascade may help resolve which processes are suitable for therapeutic targeting in all RASopathies or in a personalized, mutation-specific fashion. We show that a RASopathy mutation downstream of RAS, *Raf1*^*L613V*^, drives an increase in the number of GFAP^+^ astrocytes and OPCs. A CFC-Syndrome linked *MEK1*^*Y130C*^ mutant has recently been shown to exhibit a comparable glial phenotype [91]. *Raf1*^*L613V*^ and *Mek1*^*Y130C*^ mutations lead to enhanced ERK1/2 activity in biochemical assays, but to a lesser degree than many other RASopathy mutations [38,92,93]. *Nf1* and *Ptpn11/Shp2* mutants show altered glial properties as well, including increased GFAP labeling and OPC number [60,62,63,75]. Enhanced expression of glial markers may contribute to these results, but developing glia are known to utilize ongoing NF1 (MIM: 613113), PTPN11/SHP2, and ERK1/2 signaling to regulate glial proliferation [75,94,95]. In summary, our data further support the idea that astrocyte and OPC development is highly sensitive to upstream or downstream RASopathy mutations of varying strengths.

The precise aspect of glial development disrupted in RASopathies is not completely understood. Mutations that hyperactivate ERK1/2 signaling in neural stem cells have been shown to initiate premature gliogenesis, often at the expense of neurogenesis [6,37,96]. Recent work on Costello Syndrome-associated *H-Ras*^*G12V*^ iPSCs also detected precocious astrocyte differentiation [59]. We found little evidence that cortical neuron density was altered in *Raf1*^*L613V/wt*^ adult mice, and multiple glial markers were relatively unchanged in P14 mutant cortices. Estimates of cellular density in RASopathy mutants by our group and others often rely upon two-dimensional based approaches, which may be prone to certain technical and analytical artifacts. More rigorous systematic analysis with three-dimensional stereological methods, such as the optical fractionator or the recently developed isotropic fractionator, may be better-suited to reliably detect modest changes in cell number in samples of RASopathy brains. In the least, our data indicate the modification in the density of GFAP^+^ astrocytes and OPCs may occur during the late juvenile-young adult period in *Raf1*^*L613V/wt*^ mutants. It is unclear whether the increased glial density arises from a deficit in ongoing glial progenitor proliferation or aberrant development of progenitor pools in the SVZ. The expression of genes important for glial maturation has yet to be evaluated, such as the transient expression of Olig2 in GFAP-expressing astrocytes [97]. Finally, it will be important to examine whether the increase in glial density is cell autonomous and reversed by administration of pharmacological MEK1/2 inhibitors in adulthood.

Noonan Syndrome is typically linked to intellectual disability and other neuropsychiatric conditions that vary in severity depending on the specific mutation [9,13,20]. *RAF1*^*L613V*^ patients exhibit hallmark RASopathy features, such as hypertrophic cardiomyopathy and hypertelorism, but variable effects on intellectual capabilities that typically include impairment [39,40,41], but normal IQ [42]. However, *RAF1*^*L613V*^ individuals with increased IQ have also been reported [43]. Therefore, we asked whether *Raf1*^*L613V/wt*^ mice exhibit aberrant neurobehavioral properties. We found that *Raf1*^*L613V/wt*^ mutants had no significant deficits in measures of locomotor, anxiety, or sociability that are often disrupted in models of neurocognitive syndromes. Additional behavioral characterization using paradigms employed in other mouse models of neurocognitive syndromes will be important in future studies, such as novel object recognition, sensory gating assays, or more detailed analyses of sociability that take into account novelty-related preference. However, we did observe somewhat unexpected effects in three behavioral learning and memory assays, where mutant mice exhibited enhanced performance during specific phases. Our results show relatively improved performance of *Raf1*^*L613V/wt*^ mutants during the early acquisition, but not late, stages of the WRAM, consistent with enhanced learning during the acquisition of this task. However, mutants exhibit signs of enhanced memory following tone-cued fear conditioning, but not during the learning, or training, phase of this task. Further experimentation will be necessary to define how this mutation affects learning versus memory processes in specific circuits.

Hyperactivation of ERK1/2 has been linked to enhanced plasticity in select contexts. Costello Syndrome patients with *HRAS* mutations exhibit increased TMS-induced plasticity [98]. Moreover, *H-Ras*^*G12V*^ overexpression specifically in *CaMKII*-expressing cortical and hippocampal excitatory neurons has been shown to enhance LTP and Morris water maze performance in mice [4]. In mice exposed to different forms of behavioral stimulation, P-ERK1/2 levels transiently increase in select, heterogenous populations of cortical neurons [53,71,72,73]. It is not clear why certain neural circuits or subtypes exhibit enhanced P-ERK1/2 activation or plasticity in different contexts. Cell-specific expression of negative feedback regulators may contribute to the spatiotemporal specificity of P-ERK1/2 function. While we did not observe global changes in cortical SPRY2 or DUSP6 expression in *Raf1*^*L613V/wt*^ mice, additional cell-specific analyses are warranted. *Raf1*^*L613V/wt*^ mice provide another useful genetically-defined model to identify novel mechanisms of enhanced neural plasticity.

Our data raise important questions regarding the contributions of RASopathic non-neuronal cells to plasticity and learning. Most studies suggest the glial alterations seen in RASopathies are detrimental to nervous system function. GFAP upregulation is a hallmark sign of reactive gliosis, which is associated with multiple neuropathological conditions and observed in post-mortem *NF1* brain tissue and mouse models [60,61,62]. *H-Ras*^*G12S*^-expressing astrocytes disrupt perineuronal net formation and constrain critical period plasticity [59]. Diffusion Tensor Imaging (DTI) of individuals diagnosed with a RASopathy have detected enlarged white matter tracts and aberrant myelination, presumably due to changes in oligodendrocytes, that often correlate with learning disability [64,99,100,101,102]. Oligodendrocyte-specific NF1 deletion in mice has recently been shown to drive myelin decompaction and sensory gating defects (Lopez-Juarez et al., 2018). Both astrocytes and OPCs, however, are critical for maintaining nervous system homeostasis and promoting plastic changes important for learning and memory [80,103,104,105]. Additionally, reactive gliosis is a graded response associated with specific pro-regenerative effects [106]. It seems plausible the relatively subtle biochemical effect of the *Raf1*^*L613V/wt*^ mutation may have led to mild changes in astrocyte function that minimized the negative consequences of complete activation of reactive gliosis. In support of this, we saw little change in microglial number, which often coincides with increases in GFAP labeling, and we were unable to detect a difference in perineuronal net formation in adult *Raf1*^*L613V/wt*^ forebrains. Secondly, the increase in OPC number did not lead to changes in mature myelinating oligodendrocyte density or overt deficits in myelination. Our results indicate that moderately enhanced GFAP^+^ astrocyte and OPC number is clearly not sufficient to impair learning in *Raf1*^*L613V/wt*^ mice. Additional studies of conditional, glial-specific models will be important for evaluating to what extent the *Raf1*^*L613V*^-mediated increase in glial number is involved in enhancing cognition.

The biological basis of heterogeneity between different RASopathy mutations is not completely understood [13,20,53]. Diverse levels of kinase activation between disease-linked mutations and the precise location of the mutated gene in the signaling network clearly contribute [20,93,107]. For example, studies of Noonan Syndrome-associated cardiac defects reveal that hypertrophic cardiomyopathy is observed in less than 20% of cases linked to *PTPN11/SHP2* or *SOS1* mutations [108,109], but greater than 90% of *RAF1* mutations [39,40]. Neurocognitive phenotypes tend to be more variable but ‘downstream’ mutations in BRAF, MEK1, or MEK2 generally result in more severe cognitive deficits than ‘upstream’ mutations [19,20,24,25]. Quantitative comparisons of common cellular phenotypes using IPSC-derived samples from individuals with different RASopathy mutations would be particularly useful [59,110]. Nonetheless, the variability in neurocognitive function between individuals with the same gene mutation is significant. For example, two siblings with a P491S mutation in PTPN11 have been shown to have wide variation in language ability scores; one with severe impairment, the other just below average [24]. Individuals with an L613V mutation in *RAF1* show a broad range of IQ scores that range from impairment to possibly higher than average [39,40,41,42,43]. Genetic modifiers are likely influential, but difficult to identify in RASopathies due to extensive mutation heterogeneity. Further investigation of known strain-specific differences in RASopathy phenotypes provides a sensible alternative to identifying candidate modifiers [38,53]. Gene-environment interactions almost certainly modify neurocognitive outcomes but are poorly studied in the RASopathies. For example, the mildy higher basal state of astrocyte activation in *Raf1*^*L613V/wt*^ mice may render increased susceptibility to damage following exposure to environmental intoxicants or neuropathogenic viral infections.

Past studies have noted complex, sometimes paradoxical, changes in ERK1/2 activity in response to pharmacological and genetic manipulations in specific contexts. In the fly embryo, gain-of-function mutations in *Mek* induce unanticipated decreases in ERK1/2 activity in certain body segments [111]. Studies of RAF inhibitors in cancer led to the discovery of complex compensatory interactions between BRAF and RAF1 that drive a paradoxical increase in ERK1/2 activation [112,113,114]. Indeed, the same compensatory upregulation of BRAF activity contributes to ERK1/2 hyperactivation in response to select kinase-impairing *Raf1*^*D486N*^ mutations [66]. MEK1/2 inhibitors are clearly capable of reversing craniofacial and cardiac defects in *Raf1*^*L613V/wt*^ mutant mice [38]. In light of the enhanced learning and memory performance we observed in this strain, it will be interesting to examine whether pharmacological inhibitors of ERK1/2 signaling result in relative neurocognitive impairment in *Raf1*^*L613V/wt*^ mutants. Overall, our data provide further support for mutation-specific approaches to the development of RASopathy therapeutics.

## Supporting information

S1 Fig Normal patterns of P-ERK1/2 expression in *Raf1^L613V/wt^* brains. **A:** Western blots of P21 whole control and *Raf1*^*L613V/wt*^ cortical lysates showed no significant differences in the relative expression of SPRY2 or DUSP6 (mean ± SEM, n = 5). **B-C:** Control (B) and *Raf1*^*L613V/wt*^ (C) hippocampi displayed a similar pattern of p-ERK1/2 immunolabeling. Scale bar = 200 μm.
(TIF)

S2 Fig Juvenile *Raf1^L613V/wt^* cortices show normal extent of myelination. **A-D:** Representative double immunolabeled sections of P14 sensory cortex for MBP and DAPI showed no qualitative differences in the pattern of myelination between control (A, B) and mutant (C, D) cortices (scale bar = 50μm).
(TIF)

S3 Fig **A:** *Raf1^L613V/wt^* animals commit significantly fewer working memory correct errors during the acquisition phase (mean ± SEM, main effect of genotype, acquisition [F(1,32) = 8.94, p = 0.005] * = Fisher’s PLSD p < 0.05; learning [F(1,32) = 0.05, p = 0.82]; asymptotic [F(1,32) = 2.26, p = 0.14]). **B:** Evaluation of working memory correct (WMC) errors during the acquisition phase indicate a marginal interaction of trial by genotype (solid lines) (mean ± SEM, [F(2, 64) = 2.83, p = 0.07]). Individual analysis of trials 3 and 4 reveal a main effect of genotype (Trial 3: [F(1, 32) = 6.83, p < 0.05], Trial 4: [F(1, 32) = 5.82, p < 0.05]). **C:** Mutant and control animals commit comparable numbers of working memory incorrect errors (mean ± SEM, acquisition [F(1,32) = 2.04, p = 0.16]; learning [F(1,32) = 0.43, p = 0.51]; asymptotic [F(1,32) = 0.002, p = 0.98]). Similarly, we observed no differences in reference memory errors between genotypes during the testing period (mean ± SEM, acquisition [F(1,32) = 0.41, p = 0.52]; learning [F(1,32) = 0.03, p = 0.85]; asymptotic [F(1,32) = 0.17, p = 0.68]). **D:** No differences between mutant and control velocity were observed during the probe trial. **E:** Quadrant preference in the reversal probe trial is similar between mutant and control mice. **F:** No differences in spontaneous recovery are observed between control and Raf1L613V/wt animals after extinction.
(TIF)
