# Supplementary figures and images for: The Noonan Syndrome-linked Raf1L613V mutation drives increased glial number in the mouse cortex and enhanced learning
Source: PLoS Genet. 2019 Apr 24;15(4):e1008108. doi: 10.1371/journal.pgen.1008108 (PMC6502435; doi:10.1371/journal.pgen.1008108)

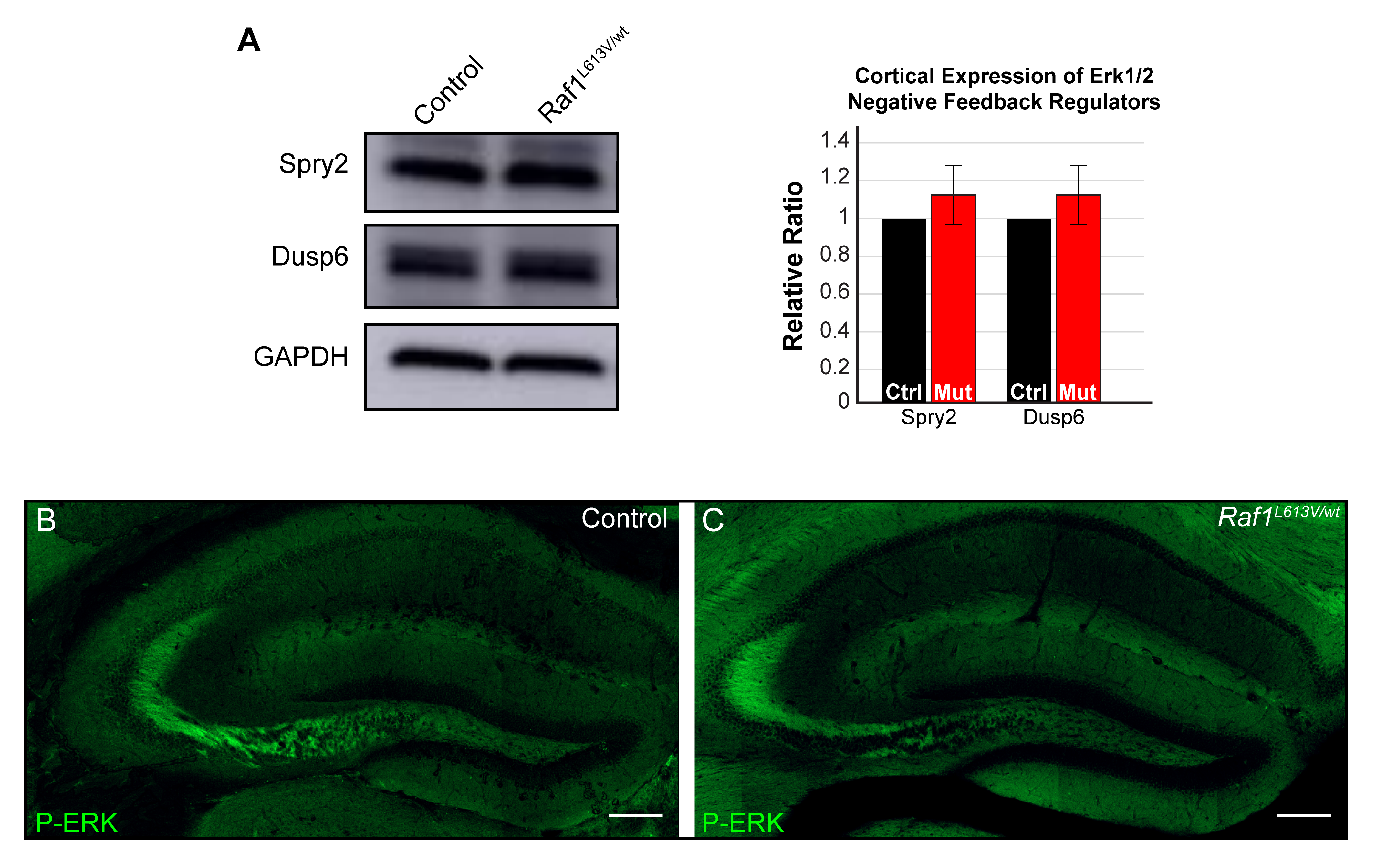

Supplement: S1 Fig — A: Western blots of P21 whole control and Raf1L613V/wt cortical lysates showed no significant differences in the relative expression of SPRY2 or DUSP6 (mean ± SEM, n = 5). B-C: Control (B) and Raf1L613V/wt (C) hippocampi displayed a similar pattern of p-ERK1/2 immunolabeling. Scale bar = 200 μm. (TIF) [file pgen.1008108.s001.tif]

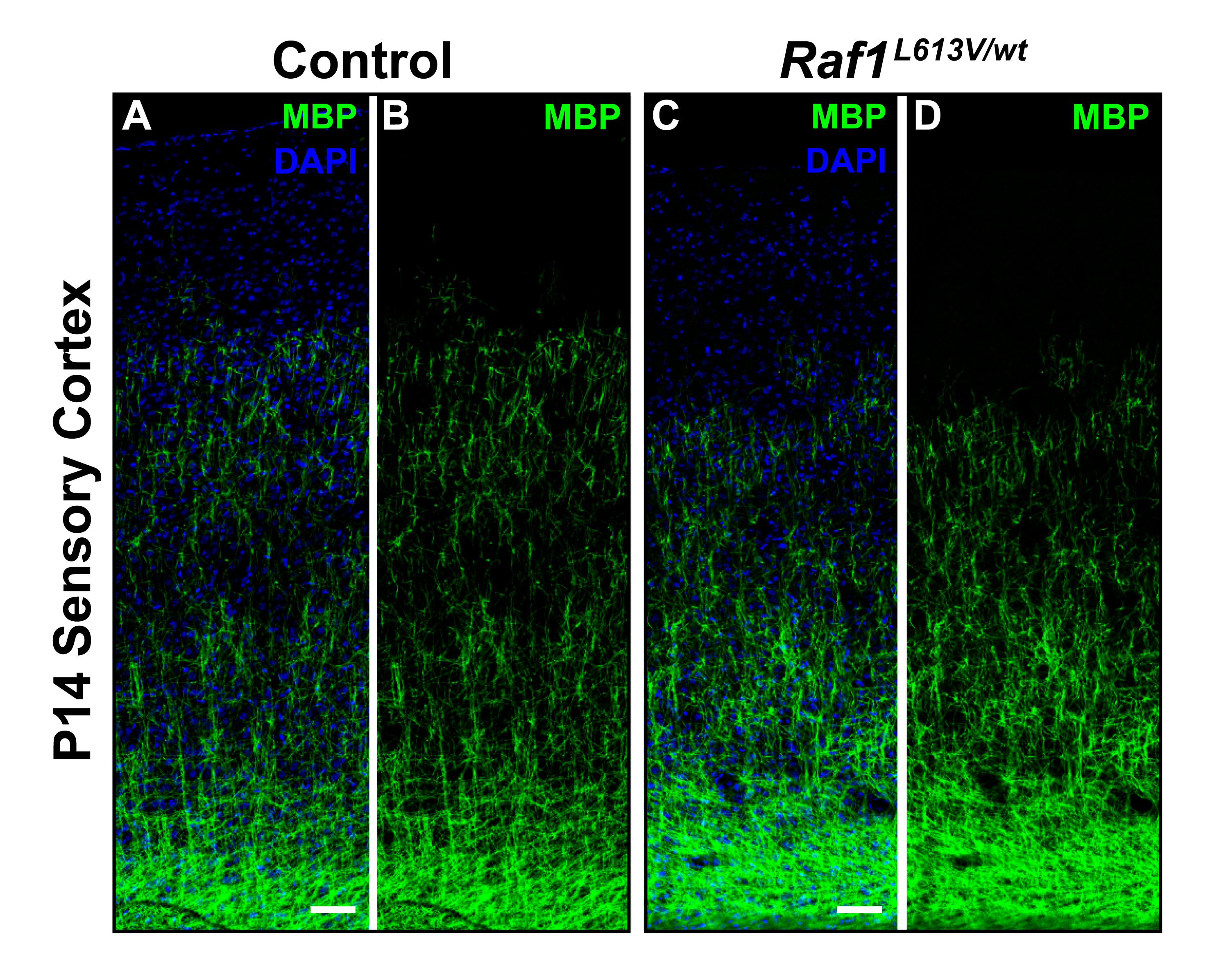

Supplement: S2 Fig — A-D: Representative double immunolabeled sections of P14 sensory cortex for MBP and DAPI showed no qualitative differences in the pattern of myelination between control (A, B) and mutant (C, D) cortices (scale bar = 50μm). (TIF) [file pgen.1008108.s002.tif]

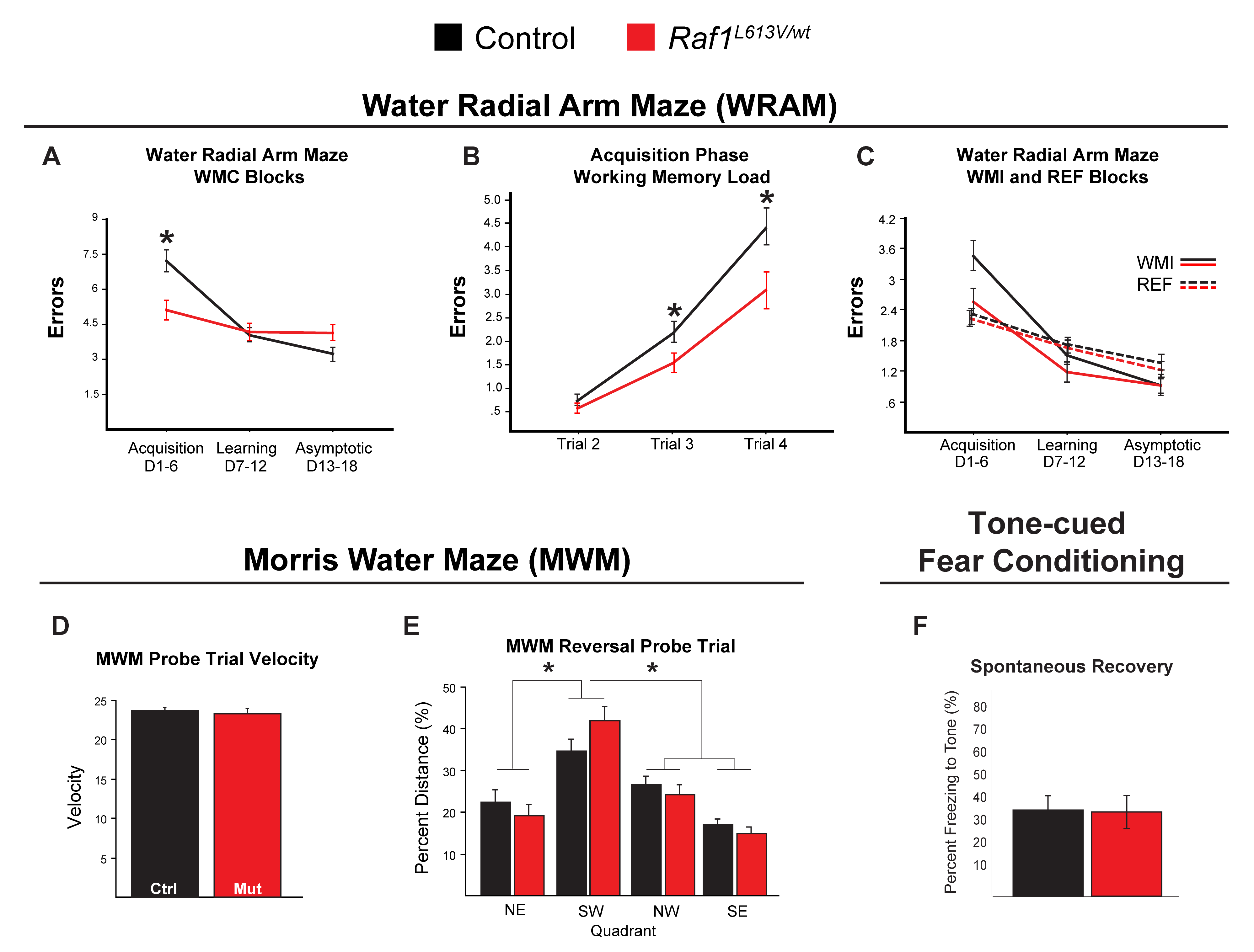

Supplement: S3 Fig — A: Raf1L613V/wt animals commit significantly fewer working memory correct errors during the acquisition phase (mean ± SEM, main effect of genotype, acquisition [F(1,32) = 8.94, p = 0.005] * = Fisher’s PLSD p < 0.05; learning [F(1,32) = 0.05, p = 0.82]; asymptotic [F(1,32) = 2.26, p = 0.14]). B: Evaluation of working memory correct (WMC) errors during the acquisition phase indicate a marginal interaction of trial by genotype (solid lines) (mean ± SEM, [F(2, 64) = 2.83, p = 0.07]). Individual analysis of trials 3 and 4 reveal a main effect of genotype (Trial 3: [F(1, 32) = 6.83, p < 0.05], Trial 4: [F(1, 32) = 5.82, p < 0.05]). C: Mutant and control animals commit comparable numbers of working memory incorrect errors (mean ± SEM, acquisition [F(1,32) = 2.04, p = 0.16]; learning [F(1,32) = 0.43, p = 0.51]; asymptotic [F(1,32) = 0.002, p = 0.98]). Similarly, we observed no differences in reference memory errors between genotypes during the testing period (mean ± SEM, acquisition [F(1,32) = 0.41, p = 0.52]; learning [F(1,32) = 0.03, p = 0.85]; asymptotic [F(1,32) = 0.17, p = 0.68]). D: No differences between mutant and control velocity were observed during the probe trial. E: Quadrant preference in the reversal probe trial is similar between mutant and control mice. F: No differences in spontaneous recovery are observed between control and Raf1L613V/wt animals after extinction. (TIF) [file pgen.1008108.s003.tif]
